# Threshold flow depths to move large boulders by the 2011 Tohoku-oki tsunami

**DOI:** 10.1038/s41598-021-92917-2

**Published:** 2021-06-28

**Authors:** Shohei Iwai, Kazuhisa Goto

**Affiliations:** 1 Department of Earth Science, Tohoku University, Sendai, 981-8578 Japan; 2 International Research Institute of Disaster Science, Tohoku University, Aoba 468-1 Aramaki, Aoba-ku, Sendai, 980-0845 Japan; 3 Present Address: Department of Earth and Planetary Science, The University of Tokyo, 7-3-1 Hongo, Tokyo, 113-0033 Japan

**Keywords:** Natural hazards, Ocean sciences

## Abstract

Around the world, numerous coastal boulders with weight of few thousand tons are suspected to have been transported by very large tsunamis, although their origins remain enigmatic. For clarifying origins of these boulders, the relation between the tsunami flow depth and the movement of meter-size boulders should be clarified but there is no proper field dataset. Here we collected first comprehensive dataset of both moved and unmoved boulders as well as the maximum flow depths along the Sanriku coast of Japan, where was affected by the 2011 Tohoku-oki tsunami based on satellite image analyses and field survey. The dataset revealed that up to ca. 1500 tons of boulders and concrete blocks were moved by the 2011 tsunami with approx. 28 m flow depth. We further revealed that most unmoved boulders were not moved because of the local setting rather than their heavy weights. The threshold of moved/unmoved boulders is estimated against the flow depth. The threshold predicted that approx. > 20 m flow depths are required to move approx. > 1000 tons boulders. The results imply that even a few thousand tons of enigmatic boulders in the world could have been moved by these sizes of the tsunami flow depths, although applicability of our results to other examples should be evaluated in the future work. We further tested the validity of an earlier proposed inverse model. Although the model result is consistent with the field observation, assumption of the appropriate parameters is problematic and further improvement of the model is required to estimate hydrodynamic features of the tsunami and to discriminate tsunami boulders from storm ones. Regarding such future work, our dataset is expected to be important for the evaluation of the improved numerical models.

## Introduction

Boulder transport by the recent tsunamis was reported after the 2004 Indian Ocean tsunami^1,2^, the 2009 South Pacific tsunami^3^, the 2010 Chilean tsunami^4^, and the 2011 Tohoku-oki tsunami^5–8^. Descriptions of such modern examples are extremely useful for elucidating the sedimentary processes of tsunami boulders and their identification criteria. In fact, discrimination of paleo-tsunami boulders from storm boulders is often problematic^3^. Sedimentological discrimination might be valid in some cases such as wide-reef environments where storm waves sufficiently attenuate during propagation on the reef. For instance, storm wave has shorter wave period than that of tsunami so that storm wave attenuates quickly. Consequently, storm boulders are distributed up to the limited distance from their original locations compared to those of the tsunami boulders^9^. However, such an approach is probably site-specific and might not be universally applicable. Moreover, because information is lacking about boulders that were not moved by tsunami waves, it remains unclear how high a flow depth of tsunami waves is necessary to transport a boulder of a certain size.

Alternatively, an inverse model is probably useful to reconstruct hydrodynamic features of waves that transported boulders and to discriminate boulders of tsunami or storm origin^10,11^. Because the input parameters for the inverse model such as volume and density of the boulder are measurable in the field, the model was widely used for identification of tsunami boulders^10^. However, the validity of the model, including assumptions about appropriate parameters, has not been confirmed based on a real-scale dataset. Therefore, whether such an inverse model is indeed useful for reconstructing flow characteristics of tsunami waves remains uncertain^12–14^. This is true simply because no dataset exists both for boulders that were moved and not moved by the recent tsunamis together with a dataset of tsunami hydrodynamic features such as flow depths. Therefore, it is uncertain whether or not the inverse model can well predict the threshold between boulders that were moved and unmoved by the tsunami.

**Figure 1:**
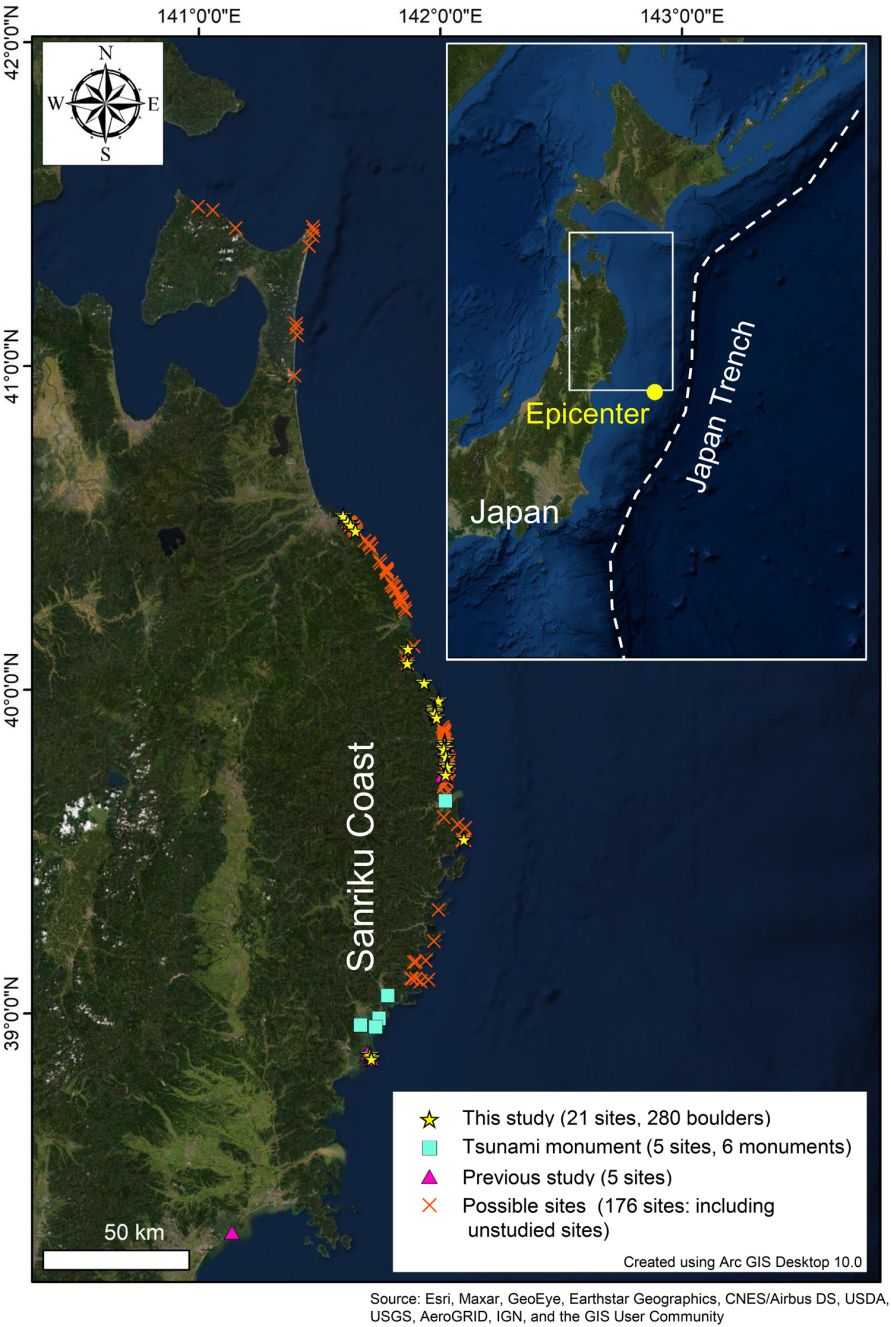
Map showing our surveyed sites, preliminarily selected sites (not surveyed), and sites studied for earlier works^5–7^. Satellite image “World Imagery with Metadata” is provided by esri atlas and the map was generated, using Arc GIS Desktop 10.0. Source: Esri, Maxar, GeoEye, Earthstar Geographics, CNES/Airbus DS, USDA, USGS, AeroGRID, IGN, and the GIS User Community.

Because tsunami events are very rare and because measurement of the positions of boulders before a forthcoming event is not easy to accomplish, satellite image analysis and complementary field surveys are probably the best methods to search for moved and unmoved boulders if both pre-tsunami and post-tsunami images are available. Regarding this point, the 2011 Tohoku-oki event is definitely the unique example because tsunami flow depths in this area were well studied. Moreover, numerous pre-tsunami and post-tsunami satellite images are available. Here we conducted satellite image analysis and field surveys of boulders along the Sanriku coast of northeastern Japan (Fig. 1), which was affected by the ca. 40 m high tsunami in 2011, to collect a dataset of boulders moved and not moved by the 2011 tsunami for testing and for future improvement of the inverse model.

### The Sanriku ria coast

Our study sites are located along the Sanriku coast on the Pacific side of northeastern Japan (Fig. 1). The area is characterized by rocky cliffs with a narrow valley in an inner bay. Small beaches and plains are situated at the entrance of the valley. Because of such a topographic setting, tsunami waves concentrate in the narrow valley and tend to record historically high run-up. In fact, the maximum run-up heights of the 2011 tsunami and the 1896 Meiji-Sanriku tsunami were, respectively, approx. 40 m and 38.2 m^15^. Numerous boulders, which were dropped off of the surrounding cliffs, are observable from the pre-tsunami satellite images and are deposited along the coast even before the 2011 tsunami. Therefore, these boulders had a chance of having been moved by the 2011 Tohoku-oki tsunami. Nevertheless, only few sites were reported as 2011 tsunami boulder fields in earlier studies^5–8^.

Some typhoons pass near the study area each year. Nevertheless, storm effects are generally slight because the area is located at high latitude (N38–N40). In fact, the highest remarkable storm wave height at northern part of Iwate Prefecture was 11.63 m in significant wave height on 2016^16^, which is far smaller than the storm waves that strike the southern part of Japan. Also, this value was measured far offshore from the coast. Considering that most of our studied coasts are within the calm inner bays surrounded by the ria coasts, storm wave should significantly attenuate before reaching to the coast.

### Boulders moved and not moved by the 2011 Tohoku-oki tsunami

Satellite images are available dating back to 1977. After 1977 but before the 2011 event, the 2010 Chilean tsunami struck the coast. However, the tsunami height at the closest point from our sites was 2.1 m^17^, which is far smaller than the height of the 2011 tsunami (ca. 40 m in run-up height). Moreover, we were unable to confirm the movement of boulders by storm waves based on a comparison of satellite images during the period without a tsunami event. This is likely because most of our survey sites were protected by the inner bay. For that reason, storm wave impacts on boulder movements are expected to be extremely limited^8,18^. Therefore, through the satellite image analysis, we expect that large boulders that clearly changed their positions before and after the 2011 tsunami were moved during this event.

**Figure 2:**
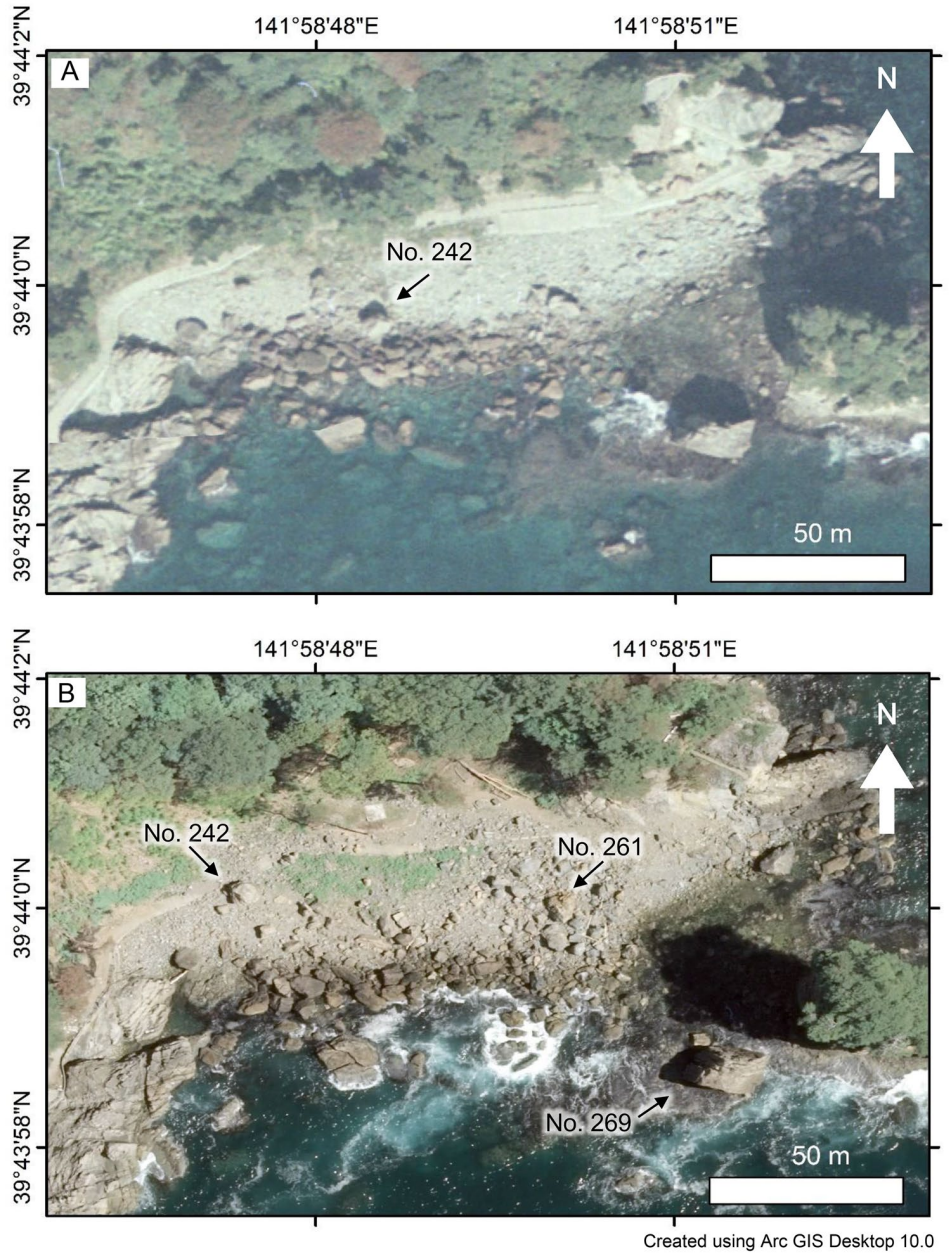
Aerial photographs, provided by Geospatial Information Authority of Japan (GSI), around “Sanno-iwa Rocks” in Miyako City, where was studied earlier by Nandasena et al.^6^, which were overlaid with using Arc GIS Desktop 10.0: (**A**) October 21, 1977 and (**B**) July 11, 2011. Numbers of boulders correspond to the Table in the Supplemental Information. No. 269 is the one in Fig. 3B. No. 242 was moved but No. 269 was not moved.

**Figure 3:**
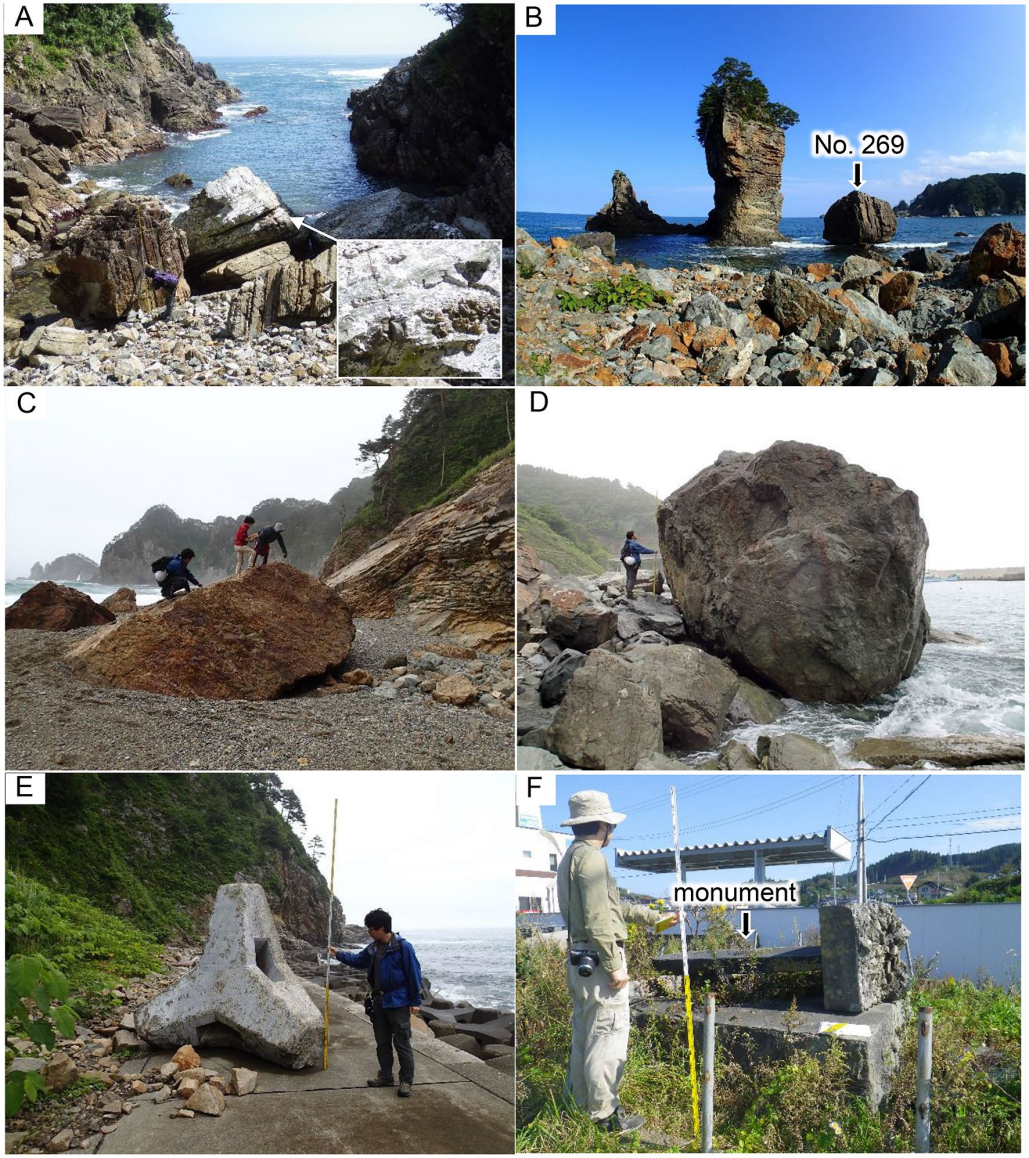
(**A**) Boulder, with remains of worms attached, deposited by the 2011 Tohoku-oki tsunami at Karakuwa Town (No. 272 of the Table in the Supplemental Information), which was studied earlier by Nandasena et al.^6^. (**B**) No. 269 boulder in Fig. 2 at Miyako City, which was not moved by the 2011 tsunami according to satellite image analysis (type 1 boulder). The boulder height is 10.4 m as a scale. (**C**) Boulder (No. 12) partially buried into the sand at Tanohata Village (type 2 boulder). (**D**) Boulder (No. 22) locked by other boulders at Miyako City (type 2 boulder). (**E**) Wave-dissipating block (No. 9) deposited on the road at Fudai Village. (**F**) Overturned tsunami monument (Iwate219) at Rikuzentakata City.

Recognizing boulders of natural rocks that were moved by the tsunami is easy if they are observable both in the pre-tsunami and post-tsunami satellite images^6^ (Fig. 2). In the field, movement of some boulders can be confirmed further based on the presence of fresh remains of attached marine organisms^6^ (Fig. 3A). However, it is noteworthy that some boulders were dropped off from the surrounding cliff by the strong ground shaking during the Mw = 9.0 earthquake by the 2011 event. Because such boulders were not observed in pre-tsunami satellite images and because there were no attached remains of marine organisms, it was not possible to confirm whether they were just dropped off or further reworked by subsequent tsunami waves. Therefore, we exclude such boulders from our study. The largest boulder of a natural rock that can be confirmed as “moved” was 10.0 × 6.6 × 5.3 m, with weight estimated as approx. 690.2 t^8^. The flow depth at the closest point was 21.9 m^19^.

Satellite images are a strong tool to identify boulders that were not moved by the tsunami (Fig. 3B). However, a field survey is mandatory because some of them were not boulders but were part of the bedrock. Boulders that were not moved by the tsunami are classifiable into two types: (1) type 1, boulders that were clearly deposited on the ground surface (Fig. 3B); and (2) type 2, boulders that were partially buried in the beach sand (Fig. 3C), and/or locked by other boulders (Fig. 3D) or located immediately in front of a high cliff.

Movement of artificial blocks such as wave-dissipating concrete blocks (Fig. 3E) and tsunami monuments (Fig. 3F) were easily recognized both in the satellite images and field observations together with eyewitness accounts. The largest artificial blocks are concrete blocks at the Aneyoshi site reported by Sugawara et al.^20^, the weight of which is estimated in this study as approx. 1462 t. This one is the heaviest in our study area. The flow depth at the closest point was 27.6 m^15^.

We also studied movement of tsunami monuments, which were constructed far inland near the inundation limit lines of historical tsunamis in 1896 and 1933^21^. They weighed several tons, with flow depths at respective sites of generally a few meters with up to 11.8 m. Although these monuments were cemented on the foundation stone, some were indeed overturned. The effects of agglutination might therefore be small compared to the devastating tsunami wave energy.

Among 280 studied boulders, movement or lack of movement of 21 boulders were uncertain because they are potentially bedrock or are not measurable because of their inaccessible locations. Therefore, further discussion will be made for 259 boulders and 6 monuments by excluding 21 uncertain ones.

### Relation between boulder weight and tsunami flow depth

**Figure 4:**
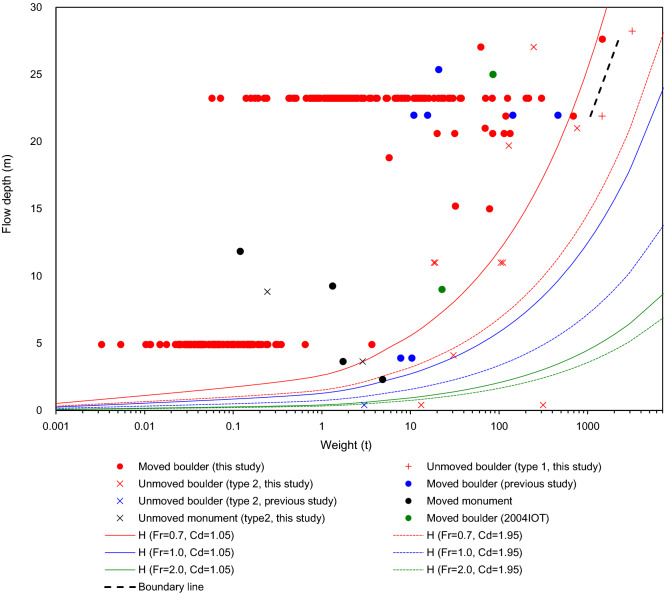
Diagram showing the relation between the boulder weight (tons) and flow depth (m).

Weights of all boulders are shown against nearby flow depths (Fig. 4). In Fig. 4, we additionally plotted 8 boulders moved and unmoved by the 2011 Tohoku-oki tsunami based on previous works^5–7^. The figure clarifies that light boulders tended to be moved, especially with deep flow depths. However, a transitional zone exists with both moved and unmoved boulders, even with similar flow depths. Most of type 2 unmoved boulders are shown in this zone. Regarding unmoved boulders, type 1 boulders were not moved probably because of their heavy weights against the tsunami wave force. While, type 2 boulders were not moved, possibly because of the pre-tsunami local settings. Most unmoved boulders are classifiable as type 2. Therefore, local factors are not negligible to ascertain whether boulders were moved or not, irrespective of their sizes.

One cannot simply discuss relations between the boulder weight and the wave force acting on it if we adopt type 2 unmoved boulders because they were affected by pre-tsunami local settings. On the other hand, from field data, we can partially estimate a threshold line between moved and type 1 unmoved boulders (Fig. 4, see “Methods”), although it is uncertain how this line reaches to the point of origin because of lack of type 1 boulders with < 1000 tons. This line should divide the plot into two regions. In the left of the line, moved boulders and type 2 unmoved boulders were mixed. While, boulders in the right region of the line were probably not moved irrespective of their weights and pre-tsunami local settings. This line is therefore useful to roughly estimate the required flow depth to move a large boulder by the 2011 tsunami: approx. > 20 m flow depth is necessary to move an approx. > 1000 ton boulder. It is noteworthy that flow depths used for this study are not necessarily the depths at the time of the boulder movements. They should be regarded as maximum values. Therefore, we cannot exclude the possibility that boulders began moving with lower tsunami flow depths.

Then, to evaluate the universality of our result, we compared our dataset with other tsunami events. In Fig. 4, we also show the maximum boulders of the 2004 IOT at Lohk Nga, Indonesia (85.2 tons with 25.0 m flow depth^2,22^) and Pakarang Cape, Thailand (22.7 tons with 9.0 m flow depth^1^). It is noteworthy that no information exists for boulders unmoved by the 2004 IOT. Although tsunami characteristics and local settings differ, plots of these boulders are generally consistent with those of the 2011 Tohoku-oki tsunami. However, because flow depth data near the boulders of other tsunami events are too few, it is not possible to expand the discussion related to the variation of the plots depending on the differences of tsunami events (initial wave conditions) and local settings such as topography. Numerical modeling to estimate the flow depths at boulder sites might be useful for additional testing of the applicability of the diagram in Fig. 4.

### Testing the inverse model

The inverse model was proposed originally by Nott^10,23^. It was subsequently revised by Nandasena et al.^11^. The model simply judges whether a boulder is sliding or overturning by the hydrodynamic force of the wave: the sum of the drag, inertia, and lift forces (see “Methods” for equations). From the equations, the minimum flow velocity necessary to slide or overturn the boulder can be estimated^11^. If one can assume the Froude Number (*Fr*), the velocity can be transformed to the minimum flow depth: then it is directly comparable to the field dataset. However, the Froude Numbers of tsunami events around the coast are well known to be highly variable depending on many local factors such that no constant value can be assumed^13,14^. Matsutomi and Okamoto^24^ estimated that the Froude Numbers of recent tsunamis along the coasts varied: 0.7–2.0. This observation is supported by measurements of the 2004IOT (*Fr* = 0.61–1.04^25^) and of the 2011 tsunami (*Fr* = 1.14–1.4^26^). Therefore, here we assumed *Fr* = 0.7, 1.0, and 2.0 to draw the theoretical threshold lines among moved and unmoved boulders. To simplify the model, we ignored inertial force, which was generally regarded as rather smaller than the drag force^11^. Not only is the Froude Number a parameter with a wide range: the drag coefficient (${C}_{d}$) is also widely ranging. In fact, it has been reported as ${C}_{d}$ = 1.05^27^ and 1.95^2,28^.

Although some uncertain parameters are included, a trend of the theoretical threshold lines by the inverse model are generally consistent with the empirical line (Fig. 4). The theoretical line is highly variable depending on the assumed Froude Number and drag coefficient. Nevertheless, the empirical line generally falls within the range of the theoretical assumptions in this study (i.e., *Fr* = 0.7–2.0, *Cd* = 1.05–1.95). Our results suggest that the model concept itself should generally be fine to explain the real-scale boulder transport phenomena. Although the Froude Number should not be assumed as a constant value, it is noteworthy that the assumption of the Froude Number around 0.7–2.0 is close to field measurements of the 2011 tsunami by Nandasena et al.^26^ (${F}_{r}$ = 1.14–1.4). Indeed, a typical tsunami converts from a wave to a current after wave breaking at the nearshore zone. It can be a supercritical flow (${F}_{r}$ > 1.0) with low flow depth but high velocity around the coastal area^24,26^. Therefore, ${F}_{r}$ = 0.7–2.0 is probably a reasonable approximation. However, the validity of each parameter, including the Froude Number together with the transport mode, should be evaluated further based on field observations, water tank experiments, and numerical experiments because several parameters strongly affect estimation of the threshold lines.

### Implication for paleotsunami research

Around the world, numerous coastal boulders have origins that remain enigmatic. Some of them are extremely heavy: about 1600 tons in Tonga at 10 m elevation^29^ and 2500 tons in the Ryukyu Islands, Japan on the 12 m high cliff^30,31^. Nevertheless, no simple means exists of ascertaining whether tsunamis can indeed transport such boulders. If we accept that our diagram in Fig. 4 is generally applicable to other cases, then we infer that movements of such extremely large boulders by the tsunamis are even likely with reasonable sizes of flow depths (ca. 20–30 m), which were indeed generated during the 2011 Tohoku-oki tsunami^32^. Of course, local settings and boulder properties of such enigmatic boulders differ from the 2011 tsunami case. In addition, our diagram should be updated as a universal one in future studies by considering many factors together with many case studies of different tsunami events. Especially, identification of various size of type 1 unmoved boulders is crucially important. Also, the elevation and transport distance are expected to be other factors that can affect tsunami size estimation^31,33^. Forward modeling of boulder transport might also be useful for quantitative estimation of tsunami properties, as with the methodology proposed by Watanabe et al.^34^.

Identification of tsunami boulders from storm-driven boulders using the inverse approach might be problematic. As Watanabe et al.^13^ and Cox et al.^14^ has noted, even a storm wave can instantaneously generate a large Froude Number higher than ${F}_{r}$ = 1.0, and even reaching to ${F}_{r}$ = 2.0. It might be the same range as that of a tsunami. For the discrimination of tsunami or storm wave boulders, it would be the better way at this moment to calculate the minimum flow velocity necessary to move boulders by an inverse approach and then perform forward storm wave modeling to test whether the required flow velocity can be satisfied by a realistic scale of storm waves at the study area, as suggested by Buckley et al.^35^ and Watanabe et al.^13^.

## Methods

To select survey sites, after first inspecting satellite and aerial images before and after the 2011 Tohoku-oki tsunami, we identified boulders that had possibly been moved or not moved by the tsunami. Images are available dating back to 1977. Among 176 preliminary selected sites, we further selected 21 accessible sites (Fig. 1) including sites which were examined in earlier studies^6–8^. Field surveys were conducted during 2015 and 2018. In situ measurements were taken for positions, dimensions and densities of totally 280 boulders of natural rocks and artificial blocks. Densities were estimated using small specimens with the same type of rocks deposited near the targeted boulders. We also studied six surviving or destroyed tsunami monuments that had been constructed after the 1933 or 1896 Sanriku tsunami events to memorialize huge tsunami disasters. Their pre-tsunami positions were well mapped^36,37^. In addition, because they were generally constructed near the inundation limit of historical tsunamis (and consequently near the inundation limit of the 2011 tsunami), it is possible to evaluate movements of small boulders (monuments) by small tsunami wave energy that was well attenuated near the inundation limit. After identification of the rock types of these monuments, we assumed the likely density using the Rock Property Database (PROCK)^38^. The respective weights of boulders and monuments were then calculated based on each boulder’s volume and density.

Tsunami flow depths closest to the respective boulders were referred from data, either from The 2011 Tohoku Earthquake Tsunami Joint Survey (TTJS) Group^15^, Ministry of Land, Infrastructure and Transport^19^ or our field observation. The latter^19^ are 100 m grid data created through interpolation of numerous survey results. All data of boulders and flow depths are presented in Supplemental Information.

In order to estimate the threshold between moved boulders and type 1 unmoved boulders against flow depths, we draw the boundary line in Fig. 4. To draw this line, we used both type 1 unmoved boulders as well as the closest moved boulders in Fig. 4 that were paired up with respective type 1 unmoved boulders (Table S1). Then, we determined the midpoints between them and draw a line between these midpoints as the boundary line. Since type 1 boulders with < 1000 tons were not available, we did not extend the line toward the point of origin. It should be noted, therefore, that the line has a chance to be updated by the future works.

Following Nandasena et al.^6,11^, we assumed sliding as the initial mode of movement. Then, the hydrodynamic force acting on the boulder (with $a$-, $b$-, and $c$-axes) can be described as the sum of drag (${F}_{d}$ ), lift (${F}_{l}$), and inertia (${F}_{i}$ ) forces^10,11,23,39,40^.

1 $${F}_{d}=\frac{1}{2}{C}_{d}{\rho }_{f}(ac){u}^{2}$$

2 $${F}_{l}=\frac{1}{2}{C}_{l}{\rho }_{f}(ab){u}^{2}$$

3 $${F}_{i}={C}_{m}{\rho }_{f}(abc)\dot{u}$$

Therein, ${\rho }_{f}$ represents the water density, ${C}_{d}$ stands for drag force, $u$ denotes the flow velocity, ${C}_{l}$ is the coefficient of lift, ${C}_{m}$ denotes the coefficient of inertia, and $\dot{u}$ expresses the flow acceleration. The resistance forces, friction (${F}_{b}$) and gravity (${F}_{g}$), can be described as shown below^11^.

4 $${F}_{b}=\mu (({\rho }_{s}-{\rho }_{f})(abc)g{\cos}\theta -{F}_{l})$$

5 $${F}_{g}=({\rho }_{s}-{\rho }_{f})(abc)g{\sin}\theta $$

In those equations, $\mu$ is the coefficient of friction, ${\rho }_{s}$ stands for the boulder density, $\theta$ represents the slope gradient, and $g$ denotes gravitational acceleration. Using these equations, by assuming certain initial condition (e.g., sub-aerial or submerged settings) the minimum flow velocity necessary to slide or rotate the boulder can be calculated^10,11,23^. In fact, the velocity necessary to slide is smaller than to rotate^6,11^, so we assume that boulders start to move with slide motion.

We assume that the boulder is well inundated by the tsunami but that the flow acceleration is sufficiently small to ignore the inertia force. The boulder starts sliding if the hydrodynamic force exceeds the resistance force.

6 $${F}_{d}\ge {F}_{b}+{F}_{g}$$

Equation (6) can be transformed as shown below^6,11^.

7 $${u}^{2}\ge\frac{2\left({\rho }_{s}/{\rho }_{f}-1\right)gc(\mu {\cos} \theta +{\sin}\theta)}{{C}_{d}\left(c/b\right)+\mu {C}_{l}}$$

The coefficient of lift can be assumed as 0.178^41^. Several drag coefficients ranging from 1.05^27^ to 1.95^2,28^ were proposed. We adopted both ${C}_{d}$ = 1.05 and 1.95.

The Froude Number (${F}_{r}$) can be described as shown below.

8 $${F}_{r}=\frac{u}{\sqrt{gH}}$$

In that equation, $H$ represents the flow depth. The minimum flow velocity estimated in Eq. (7) can be transformed to the minimum flow depth necessary to move the boulder if we assume *Fr* . It was assumed for this study that *Fr* = 0.7, 1.0, and 2.0. However, in this case, $H$ might be lower than the boulder height, especially the high *Fr* cases. Therefore, we assume that tsunami waves partly inundated the boulder. The hydrodynamic force acting on the boulder in this case can be described as a sum of drag (${F}_{d}^{\prime}$) and lift (${F}_{l} ^{\prime}$) forces, by considering the inundation ratio ($\alpha$) against the boulder height.

9 $${F}_{d}^{\prime}=\frac{1}{2}{C}_{d}{\rho }_{f}(\alpha ac){u}^{2}$$

10 $${F}_{l} ^{\prime}=\frac{1}{2}{C}_{l}{\rho }_{f}(\alpha ab){u}^{2}$$

The ratio of inundation ($\alpha$) can be described as shown below.

11 $$\alpha=1(c\le H)$$

12 $$\alpha=\frac{H}{c}(0\le H\le c)$$

In this case, the resistance force, friction (${F}_{b}^{\prime}$) and gravity (${F}_{g}^{\prime}$) forces, can be described as presented in the following equations.

13 $${F}_{b}^{\prime}=\mu (({\rho }_{s}-\alpha {\rho }_{f})(abc)g{\cos}\theta -{F}_{l}^{\prime})$$

14 $${F}_{g}^{\prime}=({\rho }_{s}-\alpha {\rho }_{f})(abc)g{\sin}\theta $$

As described above, the boulder starts sliding if the hydrodynamic force exceeds the resistance force.

15 $${F}_{d}^{\prime}\ge {F}_{b}^{\prime}+{F}_{g}^{\prime}$$

When the boulder height is lower than tsunami flow depth, $\alpha$ becomes 1; Eq. (15) can correspond to Eq. (6). In Fig. 4, we assume axis of $a$ as 0.2, 1 to 15, and 20, ${\rho }_{f}$ as 1.02 t/m^3^, ${\rho }_{s}$ as 2.5 t/m^3^, $\mu$ as 0.750, and the slope gradient as 1/100. Axes of $b$ and $c$ for a certain length of the a-axis are estimated as shown below based on the empirical relation obtained from the field data found in this study.

16 $$b=0.6606a+0.00277$$

17 $$c=0.5582-0.1092$$

This inverse model can be adopted for boulders that were moved with a sliding mode, while those moved with overturning mode should be excluded. In our case, the tsunami monuments might have been overturned by the tsunami while most tsunami boulders could have been moved with a sliding mode because this mode requires the smallest wave force to move boulders^6,11^. Number of tsunami monuments are very few relative to the boulders. In order to simplify the discussion, we evaluated the inverse model by using sliding as an initial mode both for tsunami boulders and monuments.

## Supplementary Information

Supplementary Information 1.

Supplementary Information 2.

Supplementary Information 3.

Supplementary Information 4.

Supplementary Information 5.
