# Supplementary material for: Threshold flow depths to move large boulders by the 2011 Tohoku-oki tsunami
Source: Sci Rep. 2021 Jun 28;11:13434. doi: 10.1038/s41598-021-92917-2 (PMC8238949; doi:10.1038/s41598-021-92917-2)
Supplement: Supplementary file 1 — Supplementary Information 1. [file 41598_2021_92917_MOESM1_ESM.docx]

**Threshold flow depths to move large boulders by the 2011 Tohoku-oki tsunami**

**Shohei Iwai^*^**

Department of Earth Science, Tohoku University, Sendai, 981-8578, Japan, (Corresponding author, shohei.iwai.sendai@gmail.com)

**Kazuhisa Goto**

International Research Institute of Disaster Science, Tohoku University, Aoba 468-1 Aramaki, Aoba-ku Sendai 980-0845, Japan

Department of Earth and Planetary Science, The University of Tokyo, 7-3-1 Hongo, Tokyo 113-0033, Japan (present address)

1. **Dataset**

All data for the boulders and flow depths are shown in Table S1-S3. In addition, satellite images and field photographs of representative boulders are presented in Figures S1–S17.

**Figure captions**

**Figure S1.** Example of the boulder for which the origin is uncertain (No. 7). Satellite images taken on (a) September 2, 2010 and (b) on April 5, 2011 around boulder No. 7, Noda Village, provided by Google Earth. (c) Photographs of boulders in the field, (d) a cliff and cliff boulders near the coast line, and (e) a sandstone fragment with same lithofacies as boulders.

**Figure S2.** Satellite images taken on (a) June 17, 2010 and (b) on April 5, 2011 around No. 8 and No. 9, Fudai Village, provided by Google Earth. Photographs of boulders (c) No. 8 (uncertain) and (e) possible initial position of wave dissipating block of No. 9 (Fig. 3E in the main text) before the tsunami.

**Figure S3.** Satellite images taken on (a) July 20, 2009 and (b) on April 14, 2014 around No. 10, No. 11, No. 12, and No. 13, Kitayamazaki, Tanohata Village, provided by Google Earth. Photographs of boulders (c) No. 10, (d) No. 11, and (e) No. 13 in the field.

**Figure S4.** Satellite images taken on (a) July 20, 2009 and (b) on April 14, 2014 around No. 14, Raga, Tanohata Village, provided by Google Earth. Photographs of boulders (c) No. 14 and (d) No. 15 in the field.

**Figure S5**. Satellite images taken on (a) July 20, 2009 and (b) on April 14, 2014 around No. 16, Hiraiga, Tanohata Village, provided by Google Earth. (c) Photograph of boulder No. 16. (d) Long axis and height of boulders at study site. Boulder data at Numanohama tunnel and this area (No. 16) were excluded. The height of No. 16 is estimated by the regression line in (d).

**Figure S6.** Satellite images taken on (a) July 20, 2009 and (b) on April 14, 2014 around No. 17, Haipe, Tanohata Village, provided by Google Earth. (c) Photograph of the boulder in the field.

**Figure S7.** Photographs of No. 19 and No. 18. After Iwai et al. (2019).

**Figure S8.** Satellite images taken on (a) July 20, 2009 and (b) on September 1, 2016 around No. 22, No. 23, and No. 24 at Taro, Miyako City, provided by Google Earth. Photographs of boulders (c) No. 23 and (d) No. 24 in the field.

**Figure S9.** Satellite images taken on (a) July 20, 2009 and (b) on April 14, 2014 around No. 26, No. 27, and No. 28 at Settai, Taro, Miyako City, provided by Google Earth. Photographs of boulders (c) No. 26, (d) No. 27, and (e) No. 28. The area was studied previously by Nandasena et al.^6^ and Yamada et al.^7^.

**Figure S10.** Satellite images taken on (a) July 20, 2009 and (b) on April 14, 2014 around No. 29 and No. 30, Mukaishinden, Taro, Miyako City, provided by Google Earth. Photographs of boulders (c) No. 29 and (d) No. 30. (e) Close up of the No. 30 boulder that shows subaerial and submerged parts of boulder around the frame in (d).

**Figure S11.** Boulders in the tunnel (Nos. 31–153).

**Figure S12.** Photographs of boulders (a) No. 242 reported earlier by Nandasena et al.^6^ and (d) No. 261.

**Figure S13.** Satellite images taken on January 27, 2005 (a), on May 14, 2011 (b) around No. 270, Aneyoshi, Miyako City, provided by Google Earth. (c) Huge concrete block (No. 270), which was reported earlier by Sugawara et al.^20^.

**Figure S14.** Satellite images taken on (a) April 6, 2011around Kaminokura at Karakuwa Town, Kesennuma City, provided by Google Earth. (b)–(l) Photographs of boulders (Nos. 271–276). The remnant of marine organisms in (g) and (i) are, respectively, in touch with No. 274 and No. 275. (m) A boulder (No. 279) reported earlier by Nandasena et al.^6^ as a tsunami boulder, while this study assumed its origin as uncertain.

**Figure S15.** (a) Tsunami monument of Iwate 057 and (b) the close up of its base. (c) Tsunami monument of Iwate 058 and (d) its close up to show the rock type. After the tsunami, Iwate 057 has been reconstructed at the pre-tsunami location on Jodogahama coast, Iwate Prefecture.

**Figure S16.** (a) Tsunami monument of Iwate 151 and (b) close up of its base. (c) Tsunami monument of Iwate 213. (d) Tsunami monument of Miyagi 001. Tsunami monument of Iwate 151 was not moved from the initial position. Tsunami monuments of Iwate 213 was moved by the tsunami and reconstructed at the original position. Tsunami monument of Miyagi 001 was originally located at the red frame in the picture before the tsunami.

**Figure S17.** Overturned tsunami monument of Iwate 219. This was overturned at the original position.

**Figure S18.** (a) A satellite image showing the locations of tsunami monuments investigated in this study. The image was taken on December 14, 2015 and provided by Google Earth. (b) Diagram showing the relation between the weight of tsunami monuments (t) and flow depth (m). (c) Joint area between the main body and base of the tsunami monuments.
