# Supplementary material for: Threshold flow depths to move large boulders by the 2011 Tohoku-oki tsunami
Source: Sci Rep. 2021 Jun 28;11:13434. doi: 10.1038/s41598-021-92917-2 (PMC8238949; doi:10.1038/s41598-021-92917-2)
Supplement: Supplementary file 2 — Supplementary Information 2. [file 41598_2021_92917_MOESM2_ESM.pdf]

Fig.S1

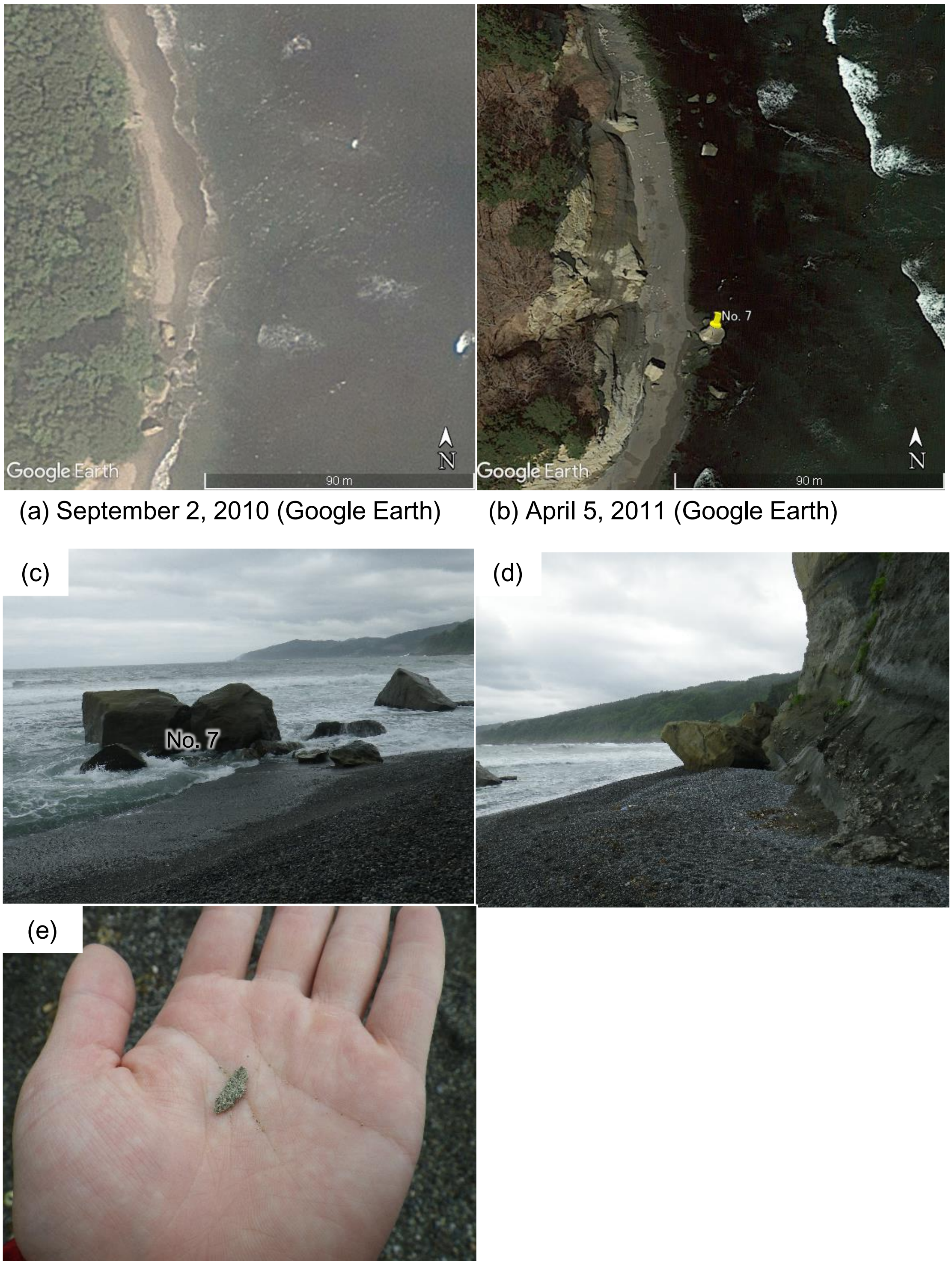

(a) September 2, 2010 (Google Earth)

(b) April 5, 2011 (Google Earth)

Fig.S2

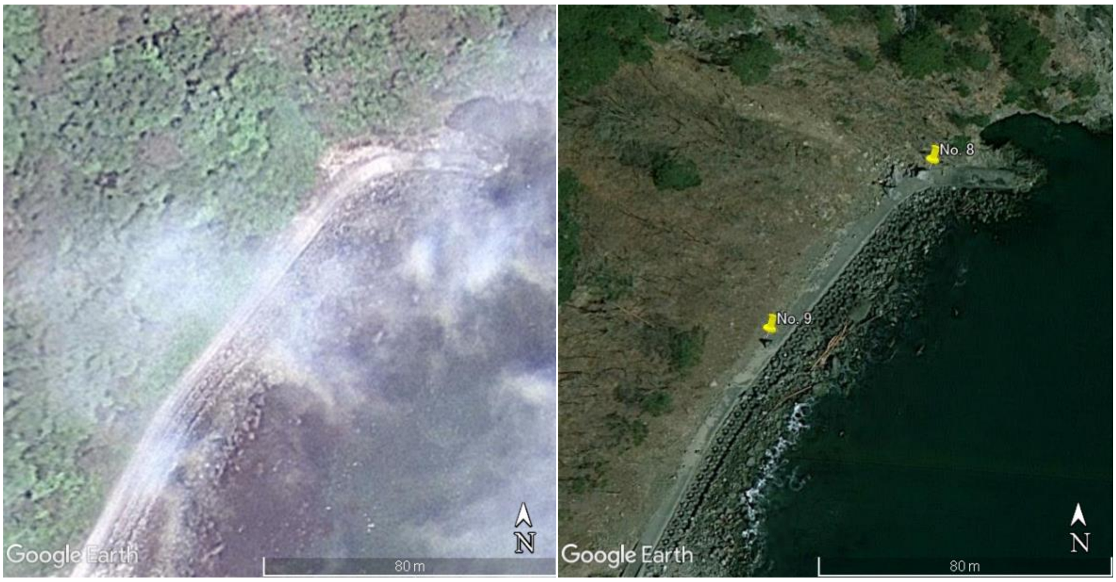

(a) June 17, 2010 (Google Earth)

(b) April 5, 2011 (Google Earth)

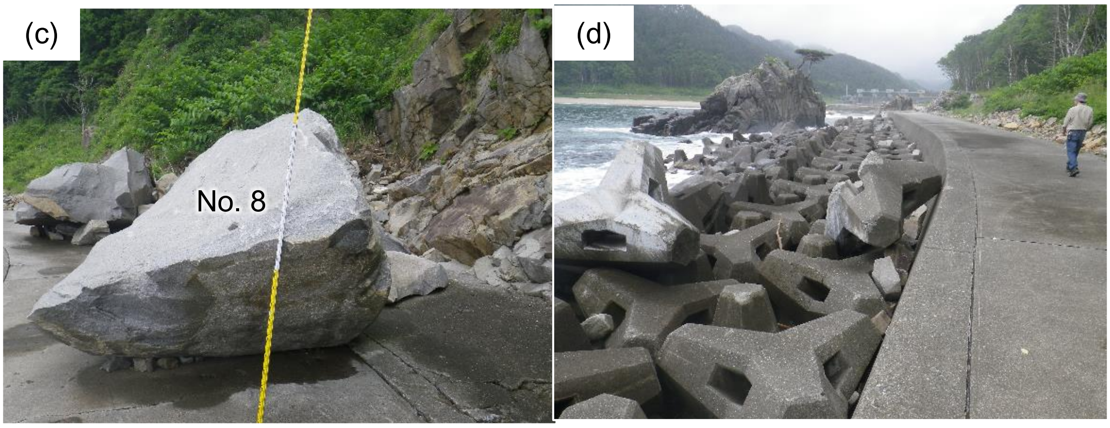

Fig.S3

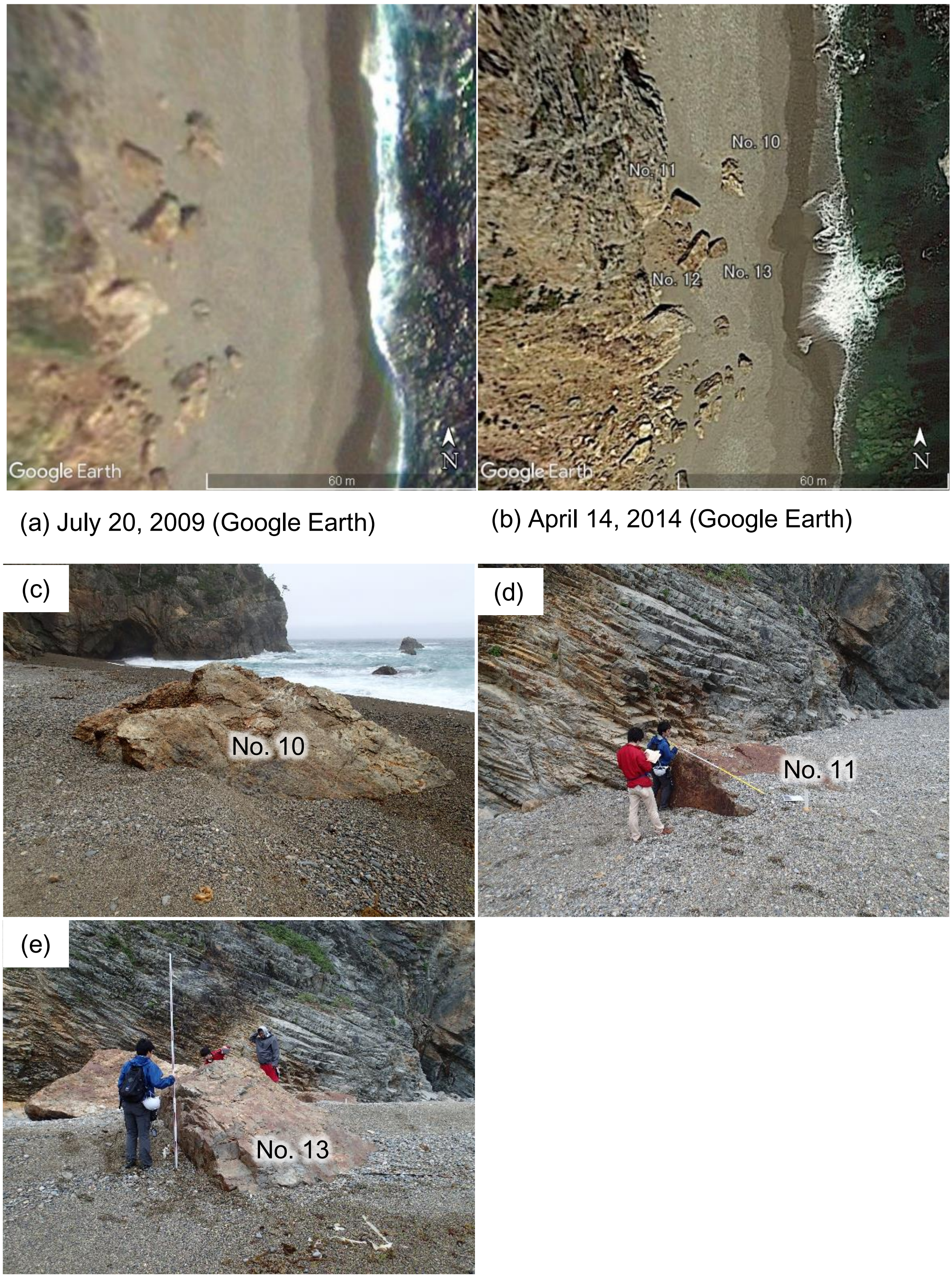
(a) July 20, 2009 (Google Earth)
(b) April 14, 2014 (Google Earth)
Google Earth
60 m
N
No. 10
No. 11
No. 12
No. 13
(c)
(d)
(e)

Fig.S4

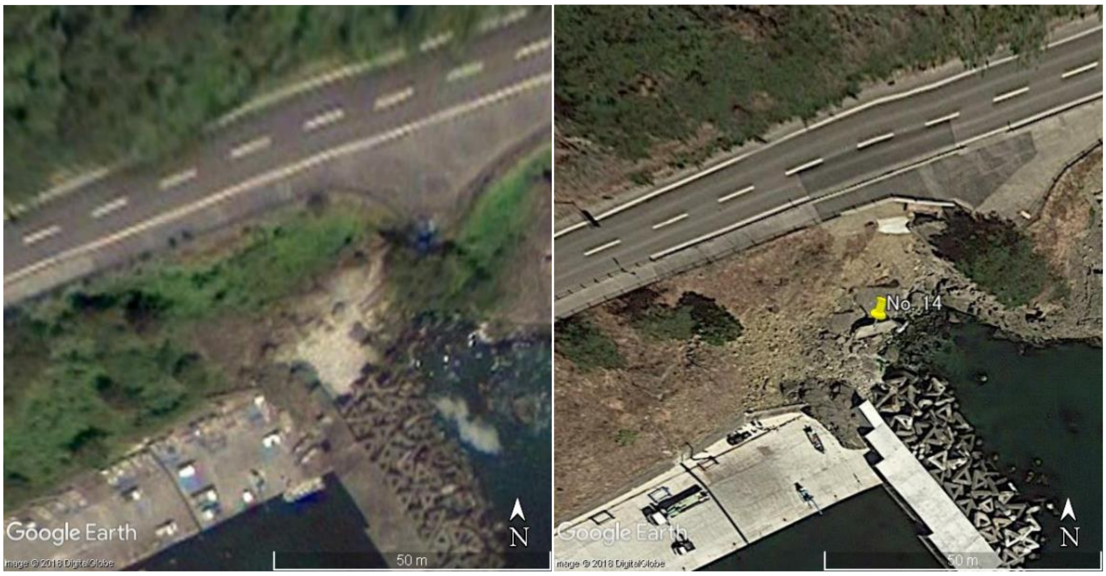

(a) July 20, 2009 (Google Earth)

(b) April 14, 2014 (Google Earth)

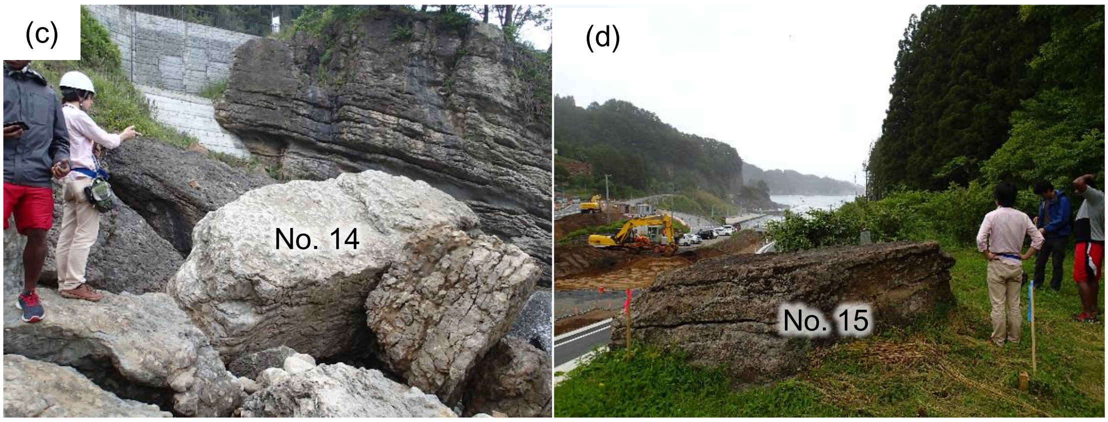

Fig.S5

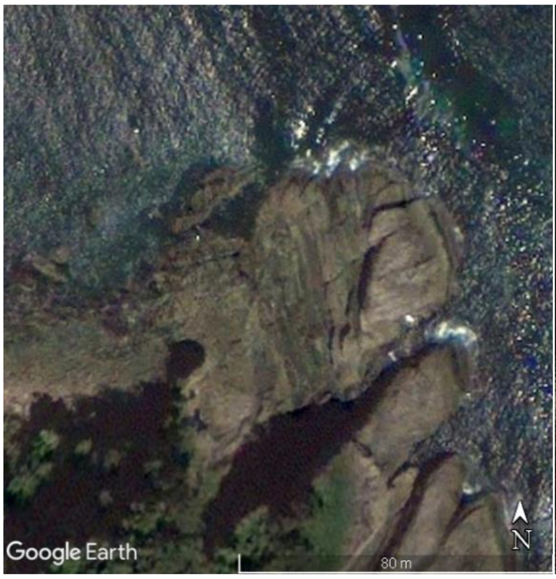

(a) July 20, 2009 (Google Earth)

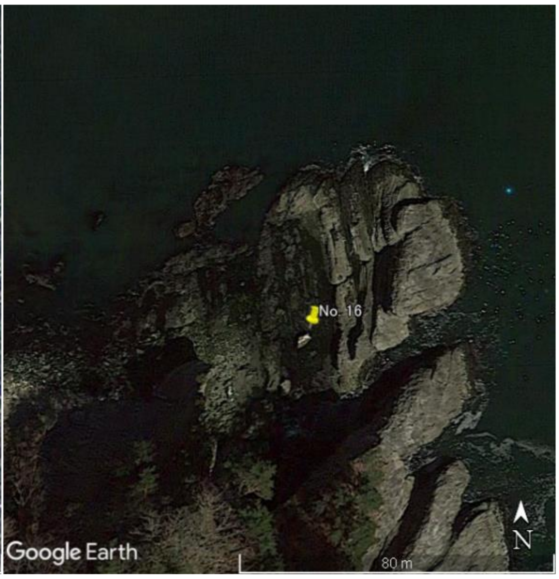

(b) April 14, 2014 (Google Earth)

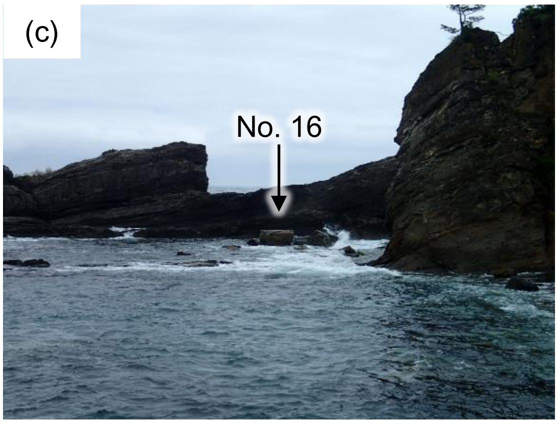

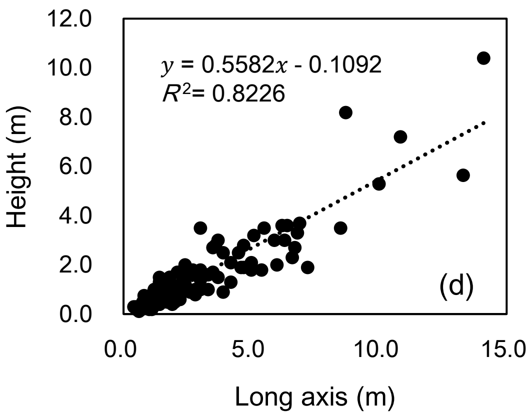

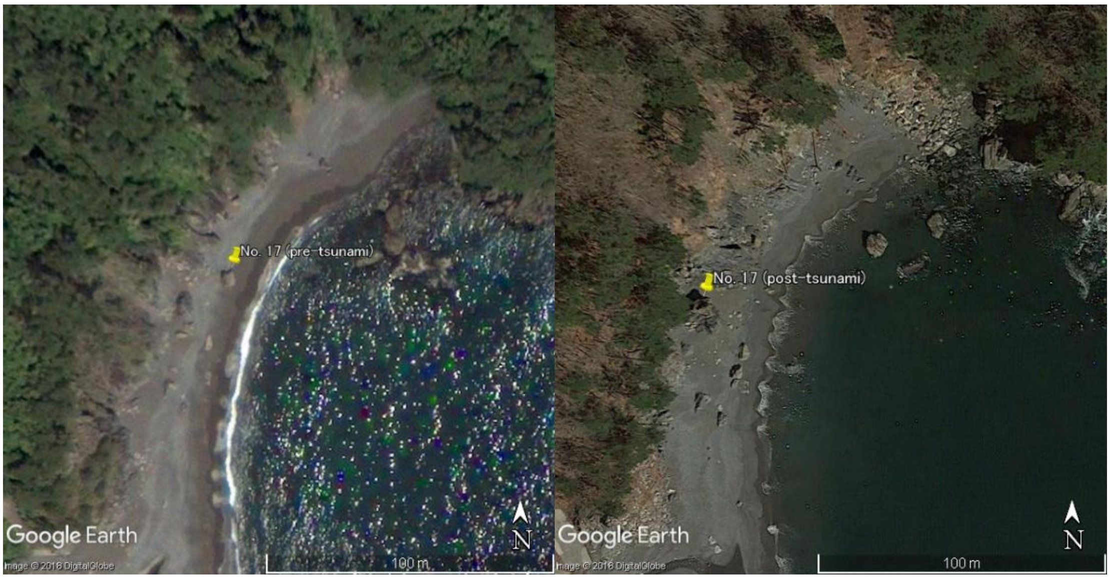

(a) July 20, 2009 (Google Earth)

(b) April 14, 2014 (Google Earth)

(c)

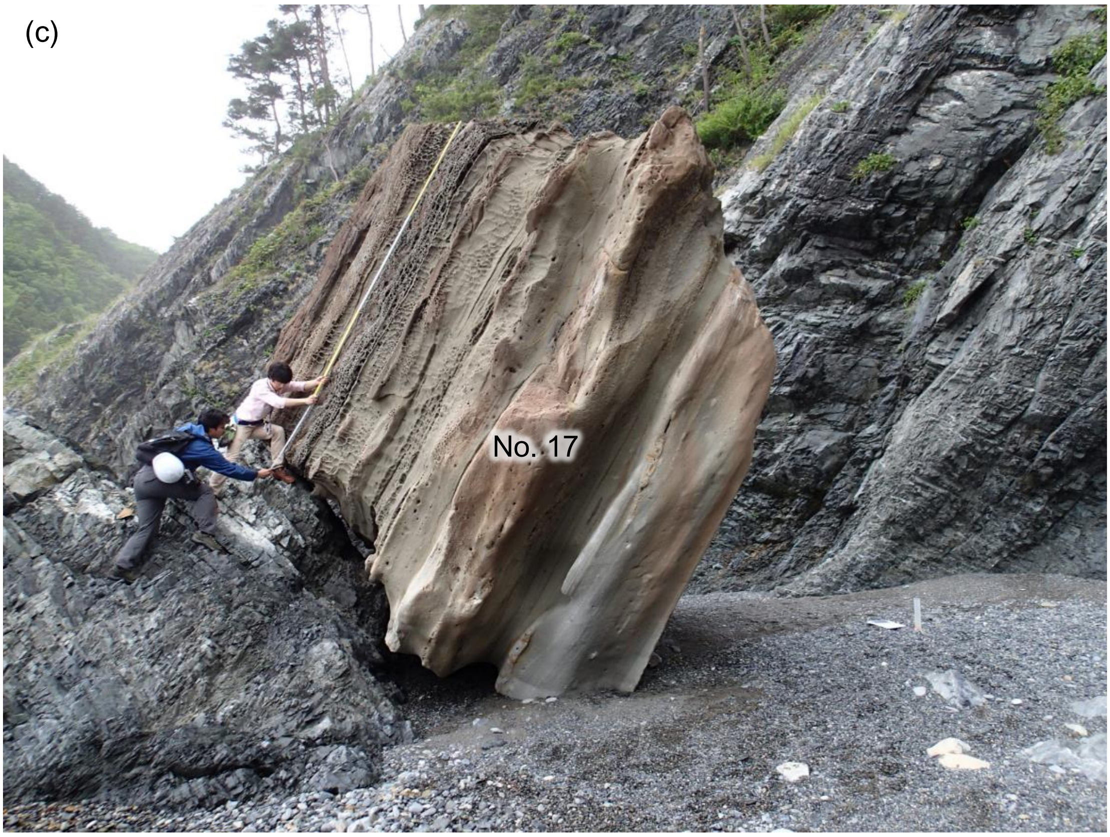

Fig.S7

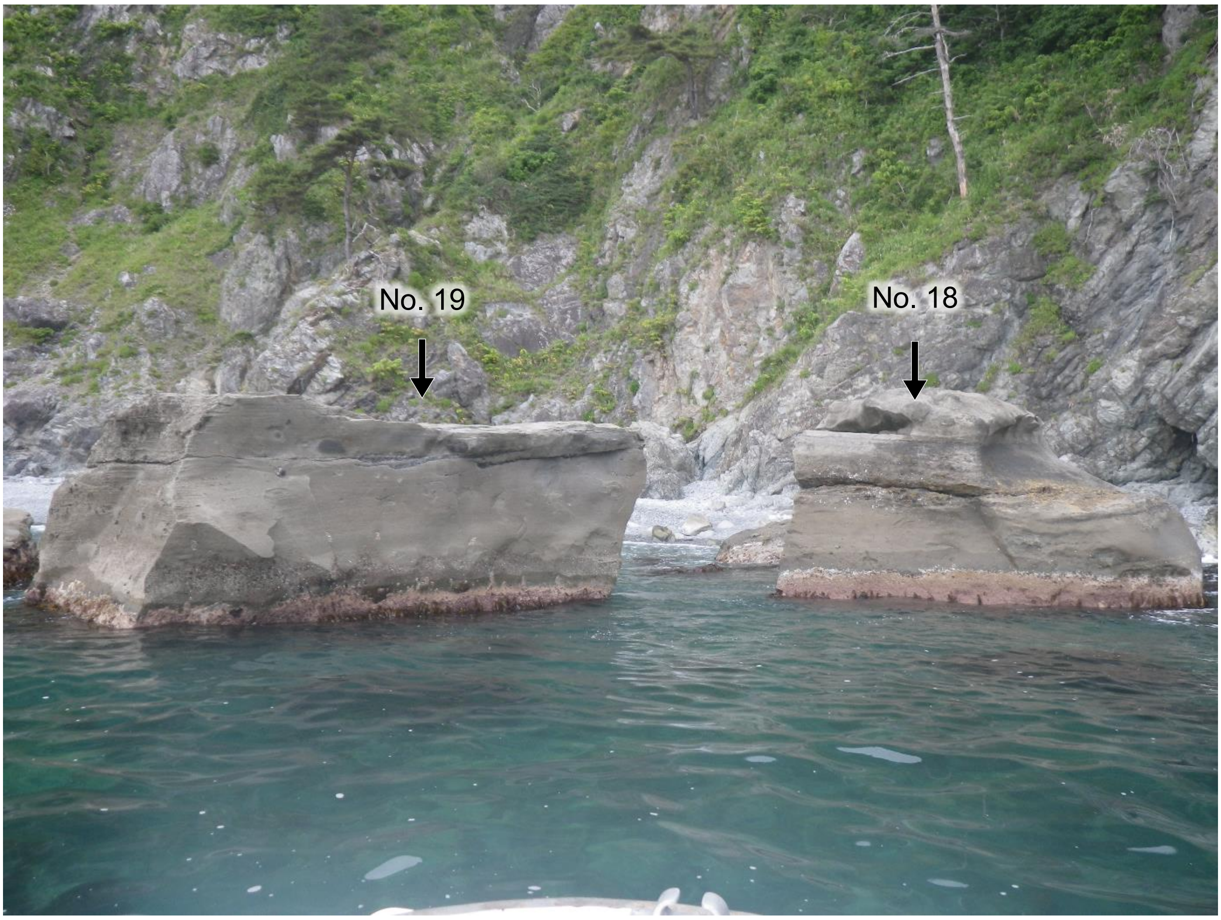

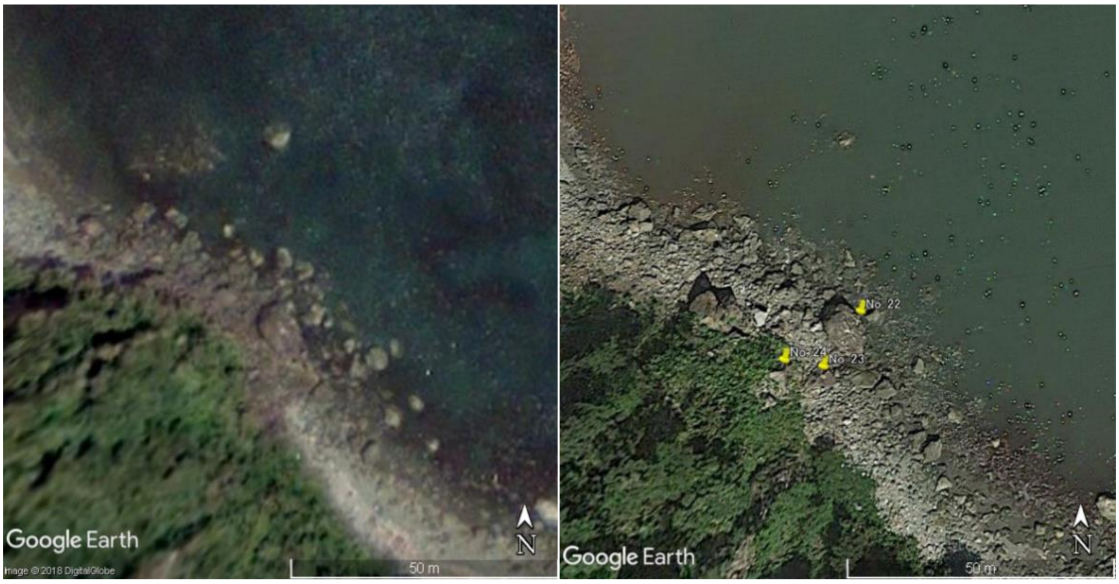

(a) July 20, 2009 (Google Earth)   (b) September 1, 2016 (Google Earth)

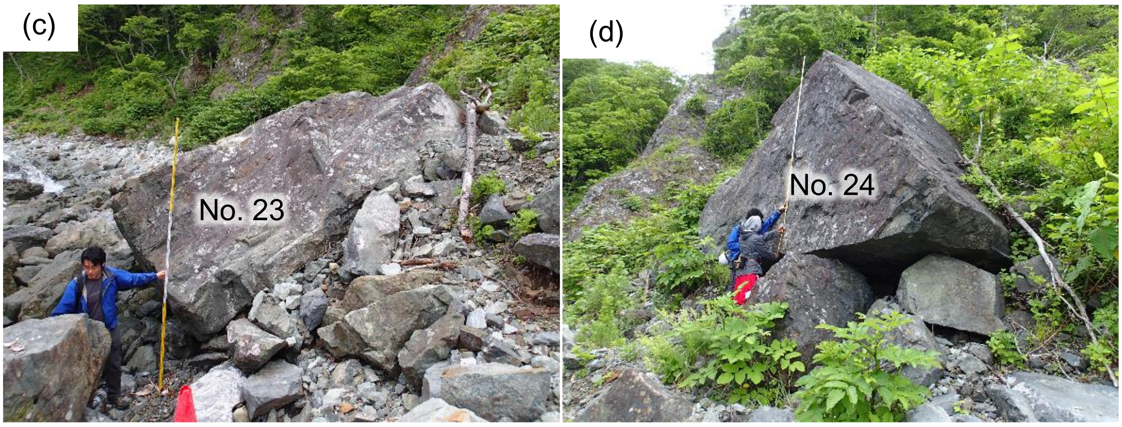

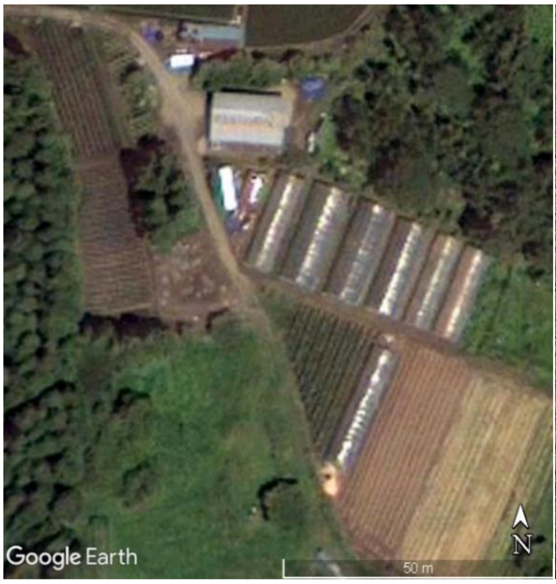

(a) July 20, 2009 (Google Earth)

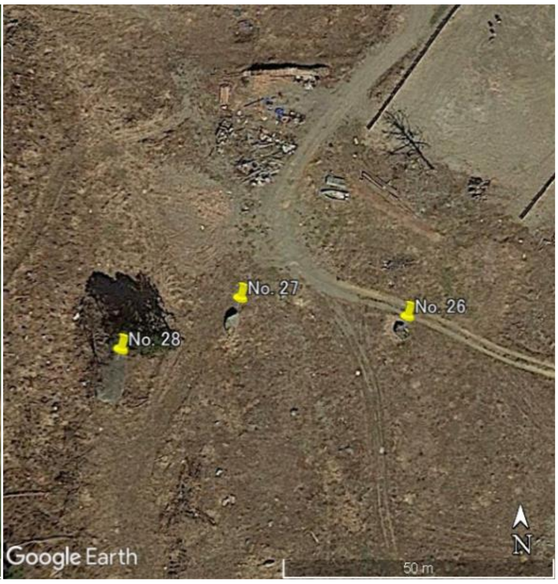

(b) April 14, 2014 (Google Earth)

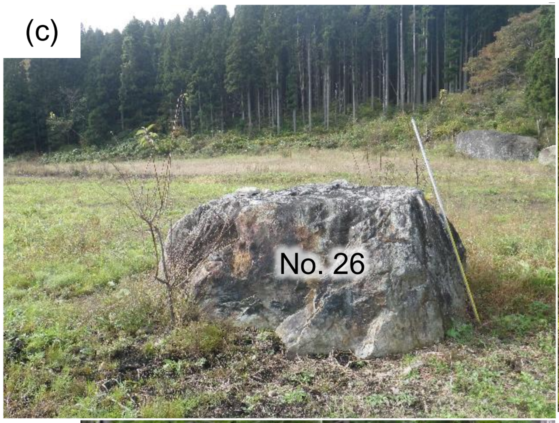

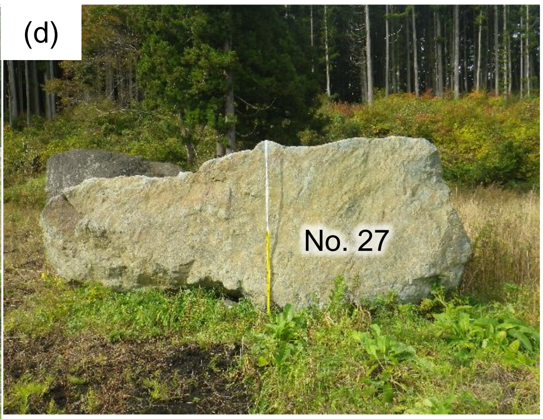

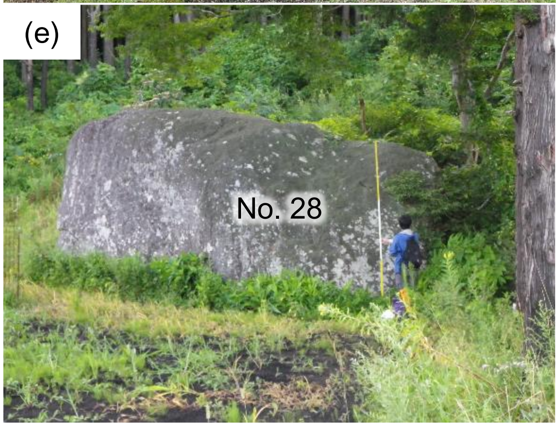

Fig.S10

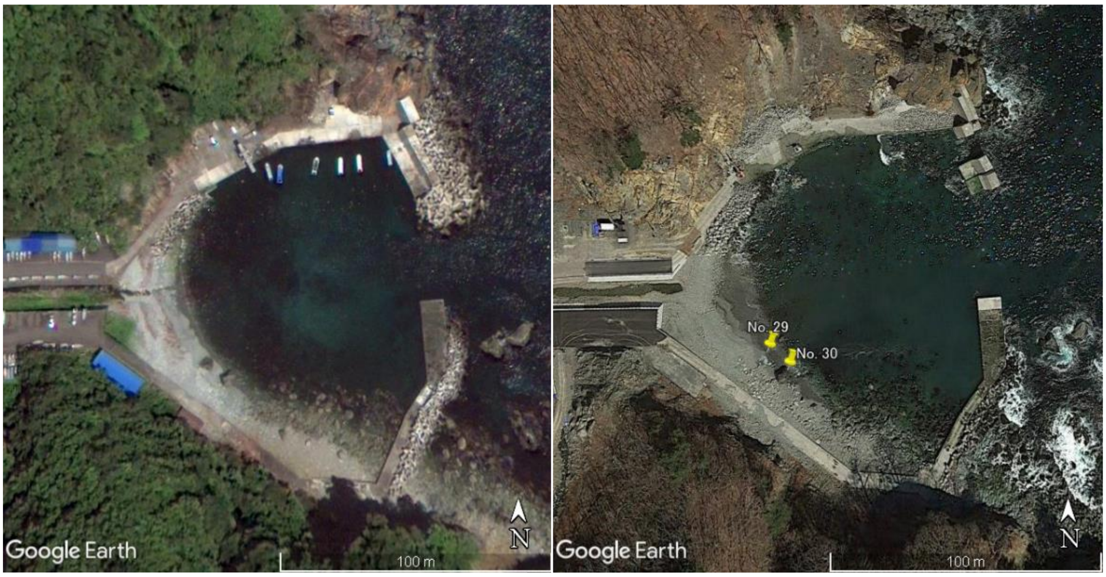

(a) July 20, 2009 (Google Earth)

(b) April 14, 2014 (Google Earth)

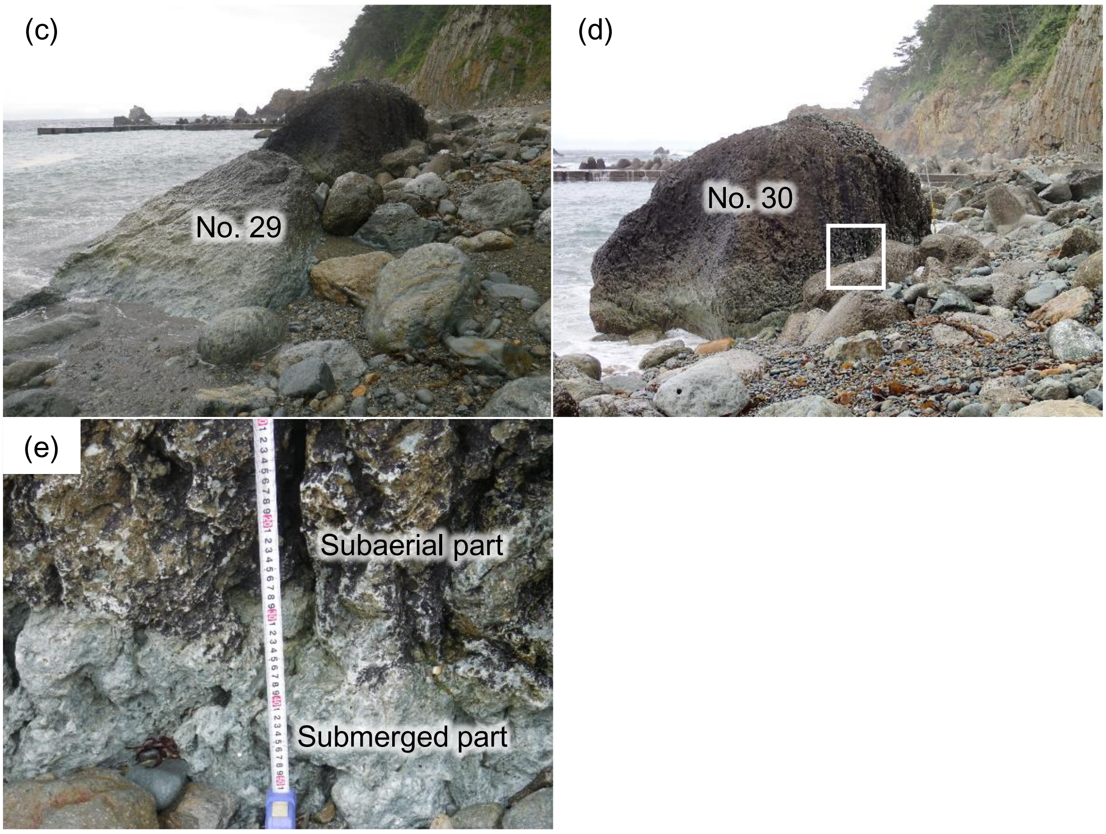

Fig.S11

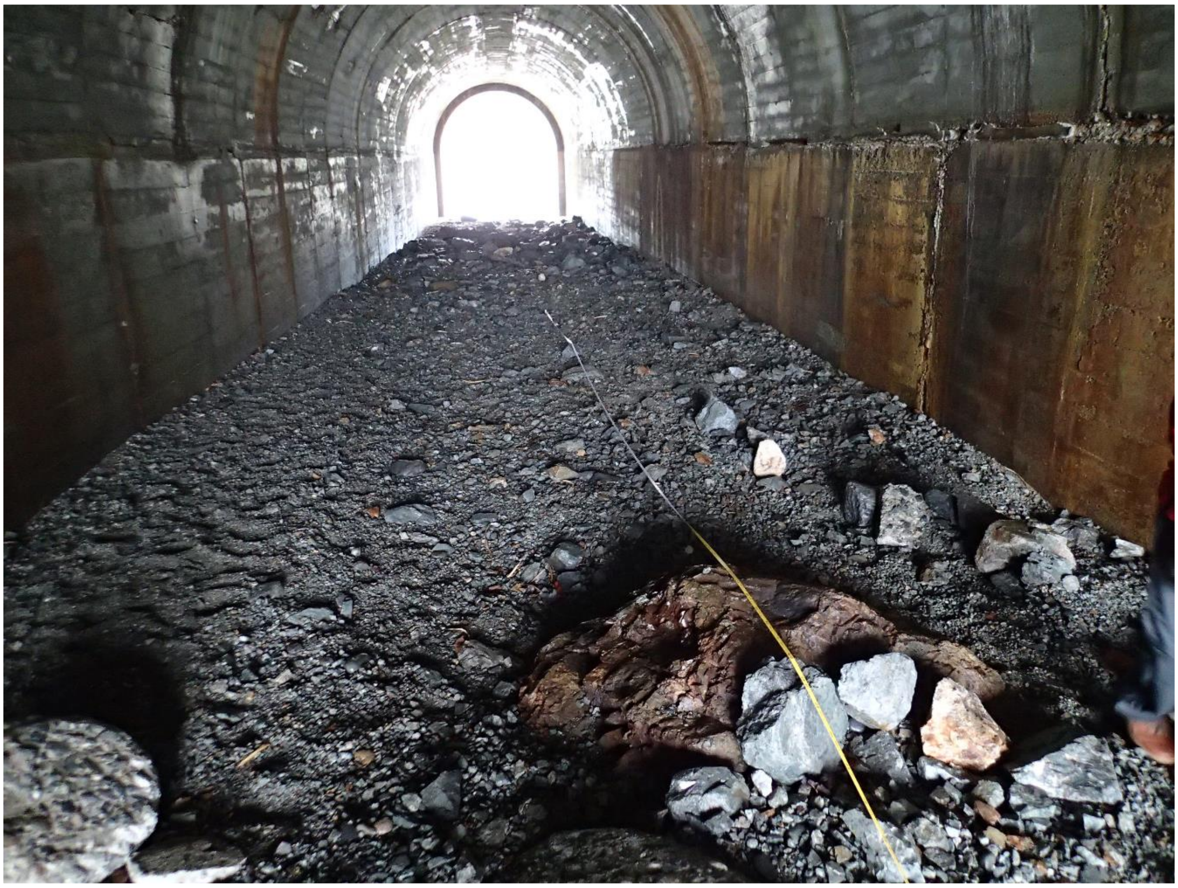

Fig. S12

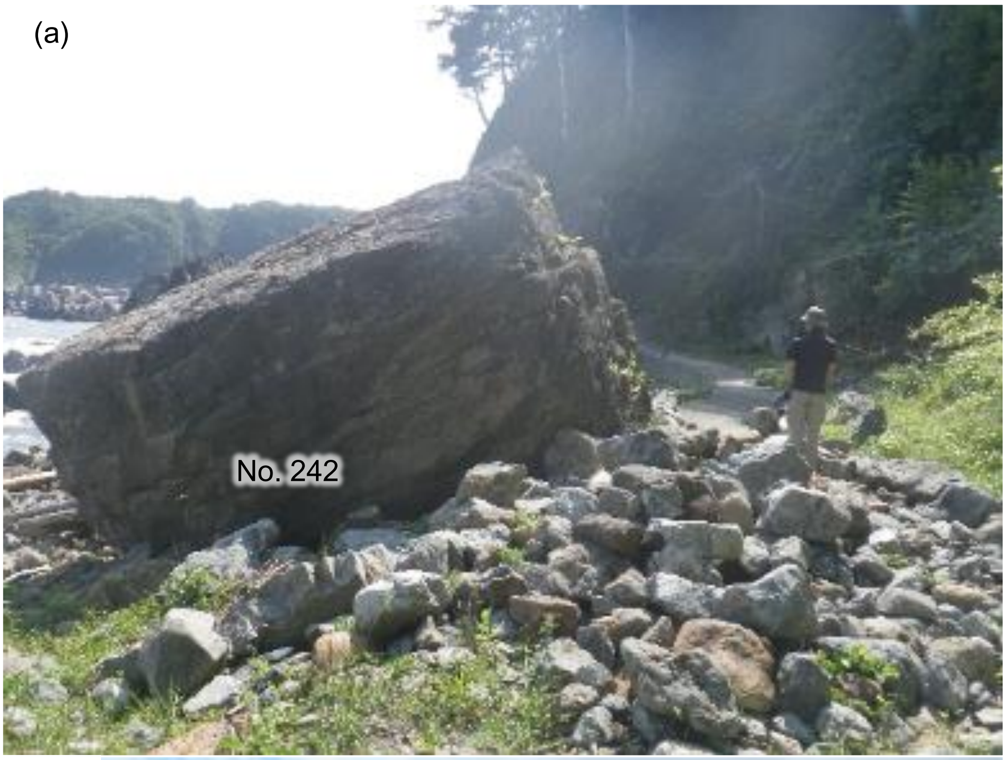

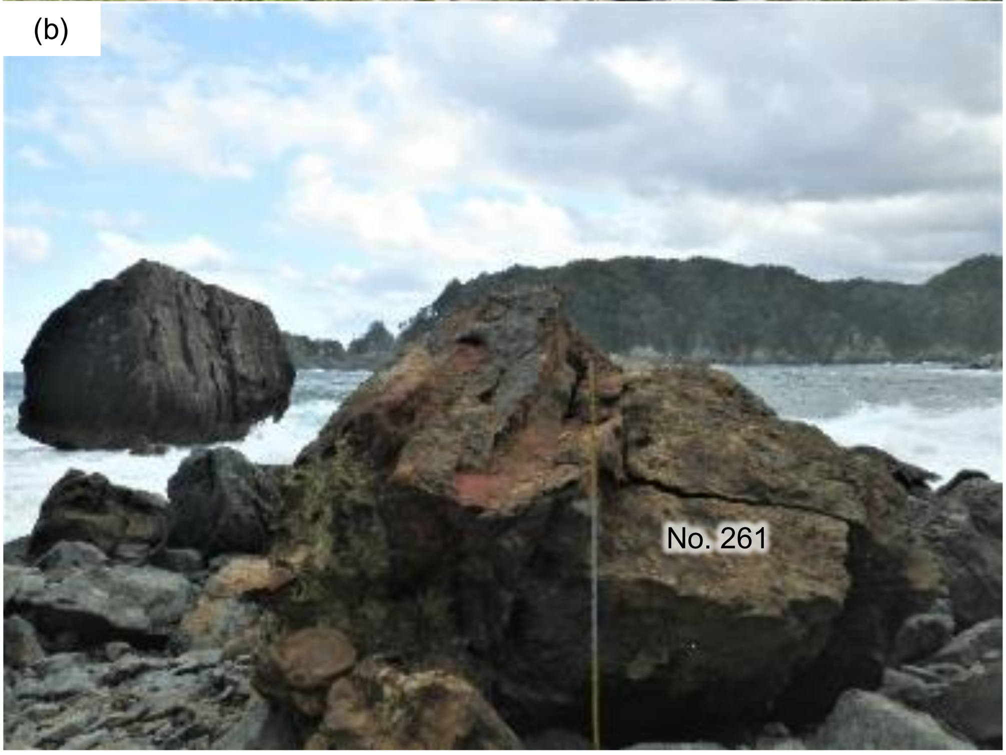

Fig. S13

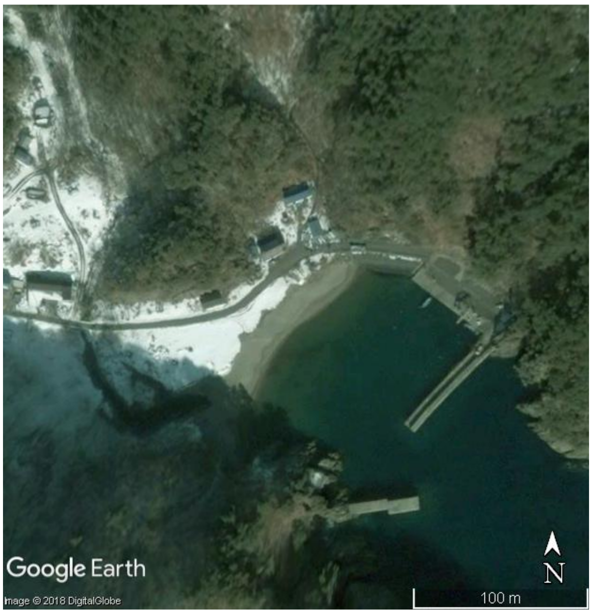

(a) January 27, 2005 (Google Earth)

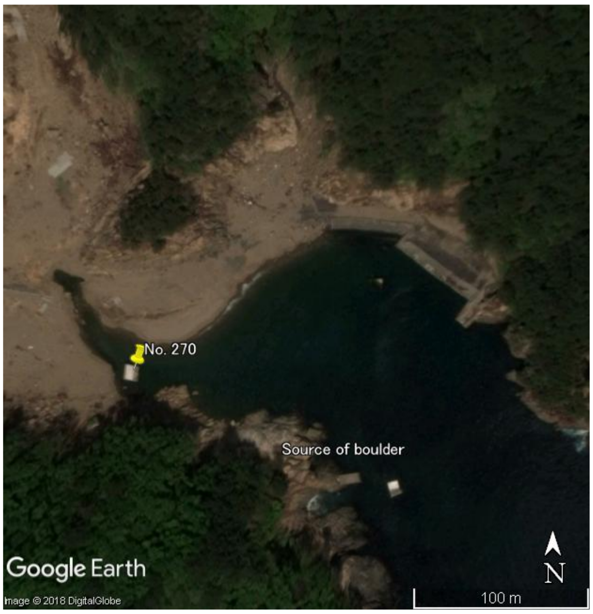

(b) May 14, 2011 (Google Earth)

(c)

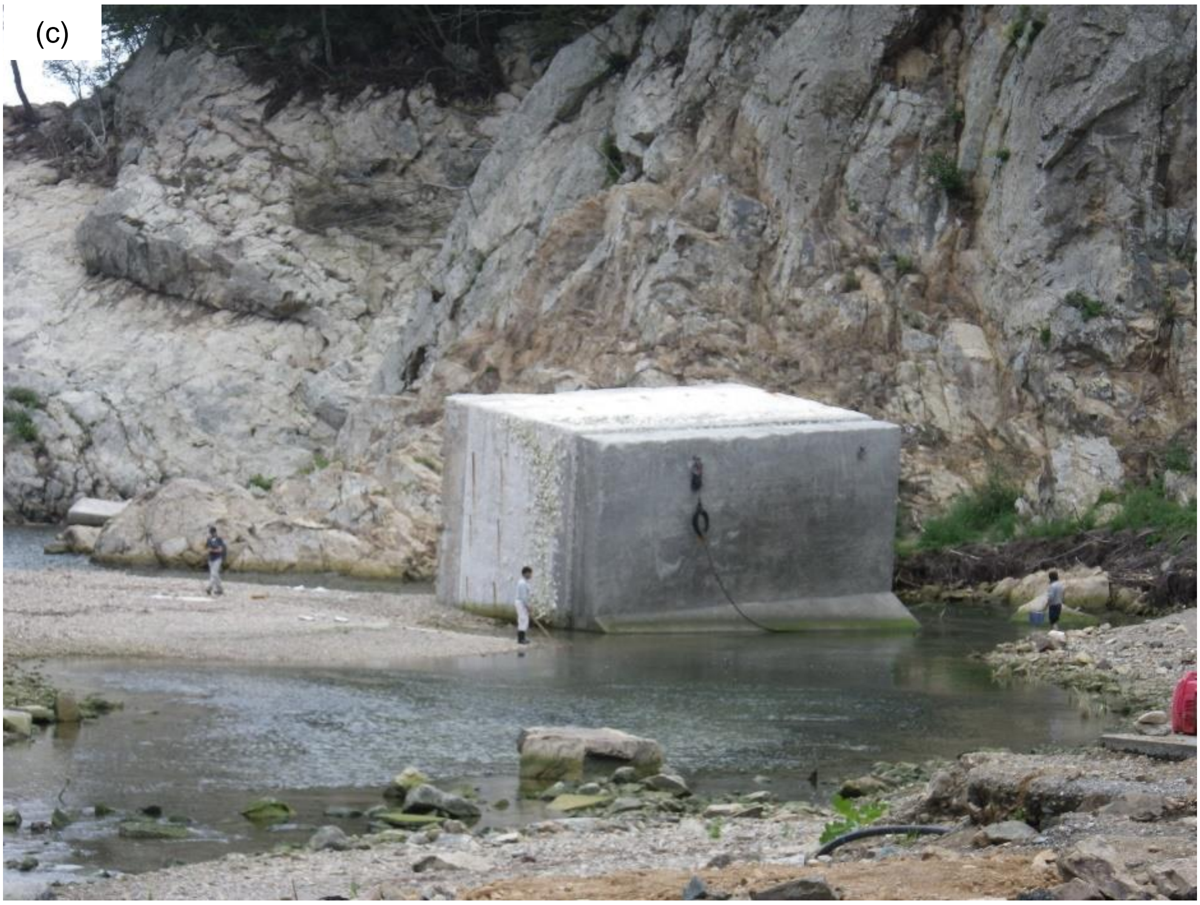

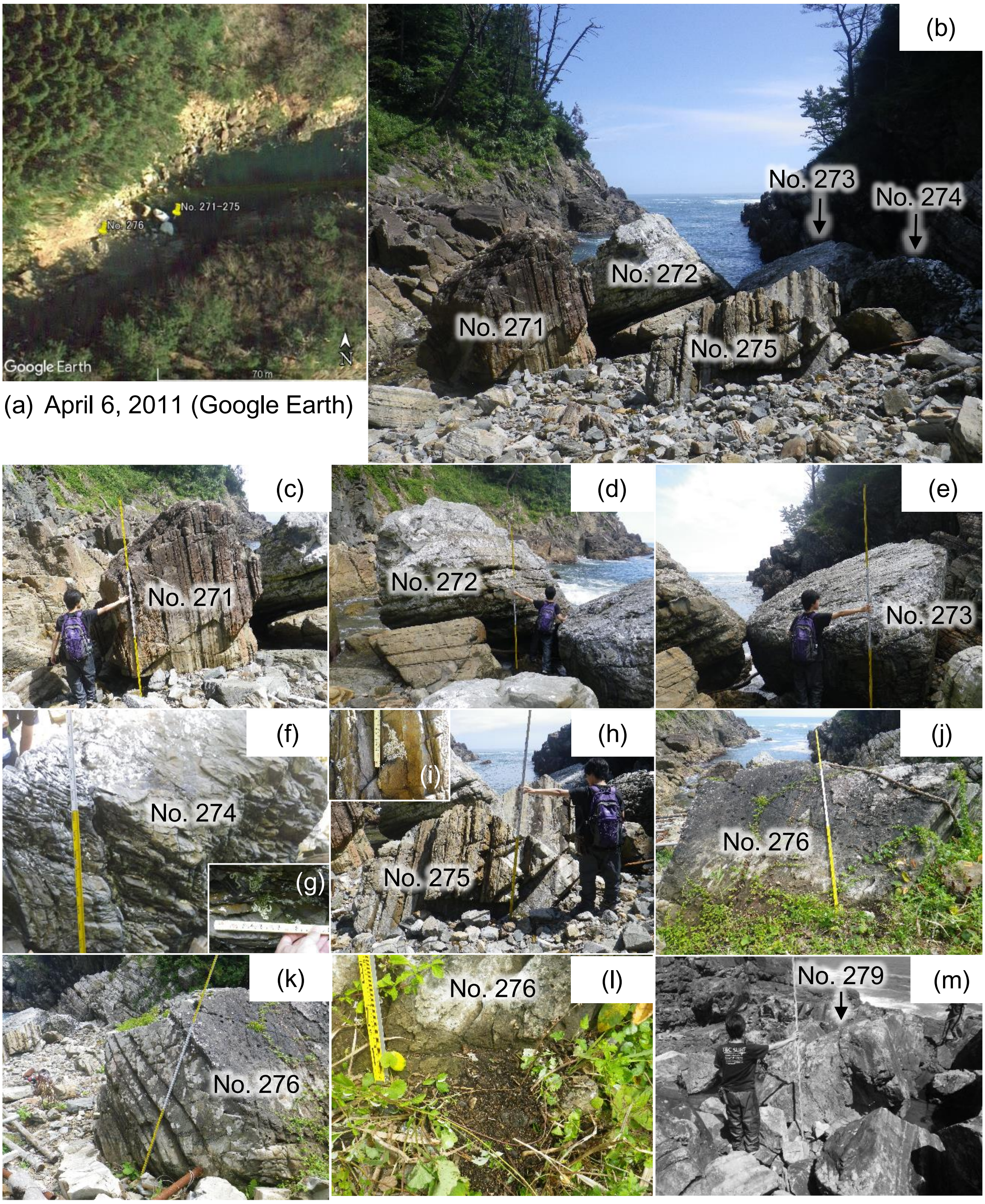
No. 271-275
No. 276
Google Earth
70 m
N
(a) April 6, 2011 (Google Earth)
(b)
No. 273
No. 274
No. 272
No. 271
No. 275
(c)
No. 271
(d)
No. 272
(e)
No. 273
(f)
No. 274
(g)
(i)
(h)
No. 275
(j)
No. 276
(k)
No. 276
(l)
No. 276
(m)
No. 279

Fig.S15

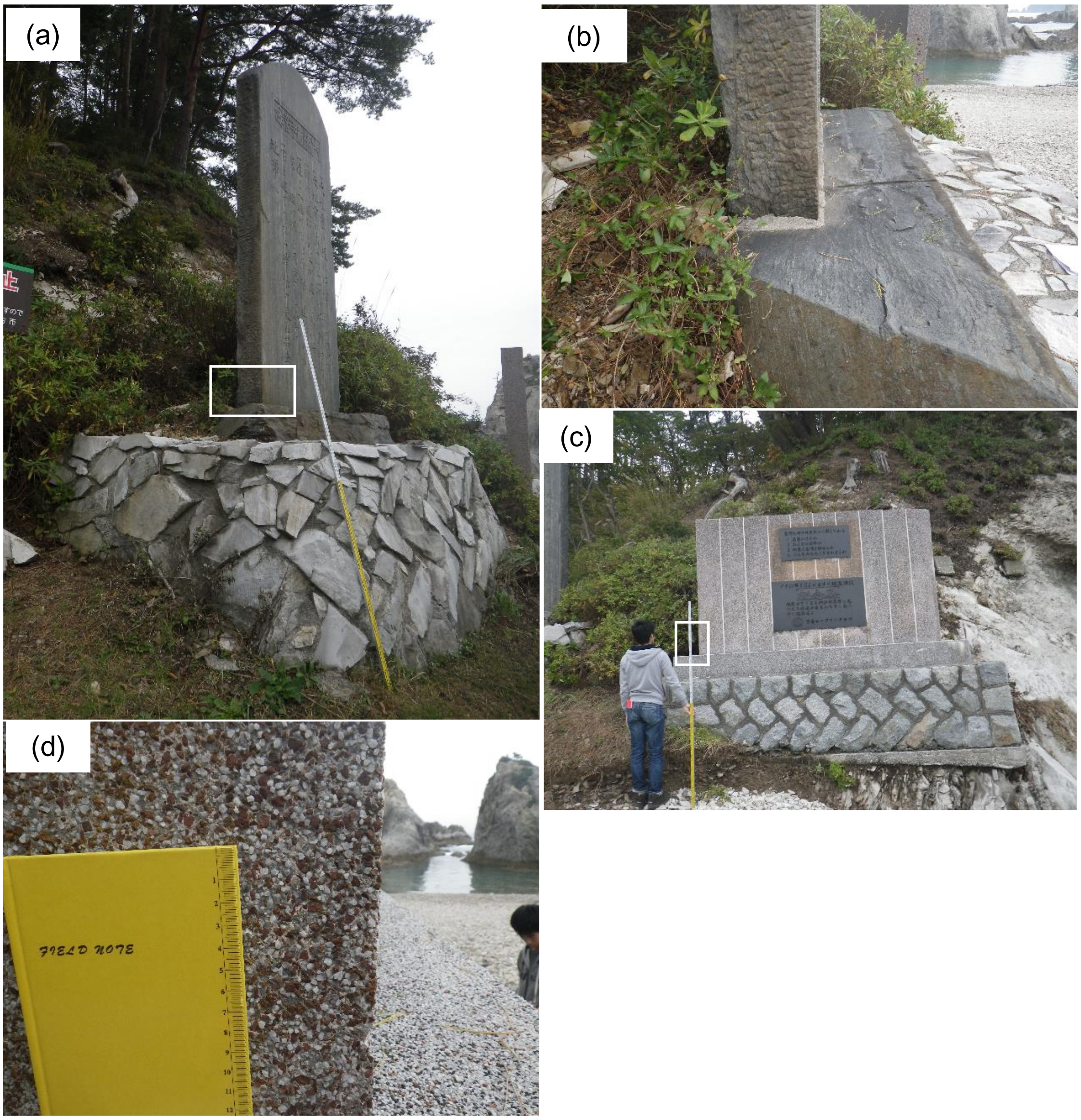

Fig.S16

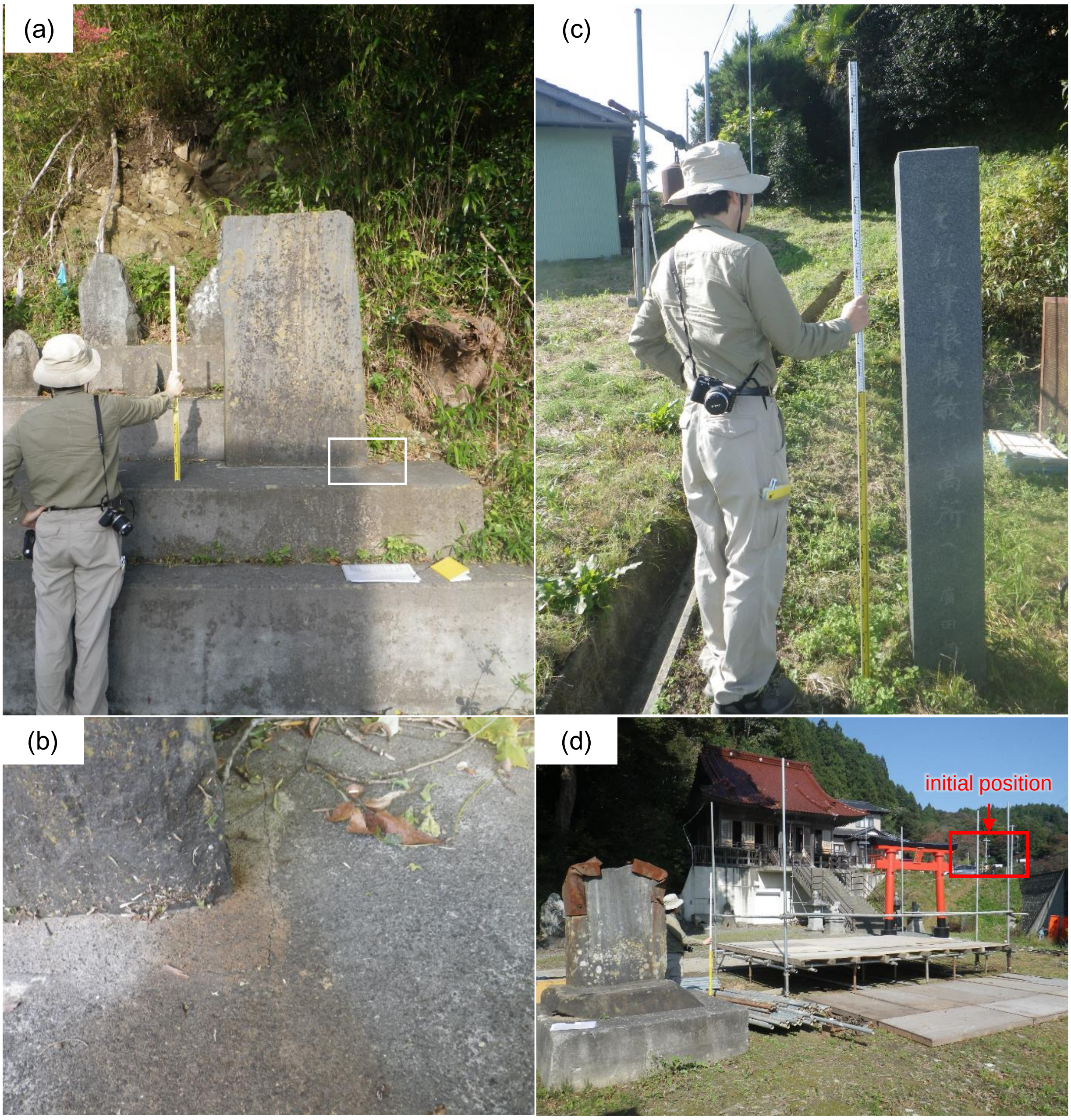

Fig.S17

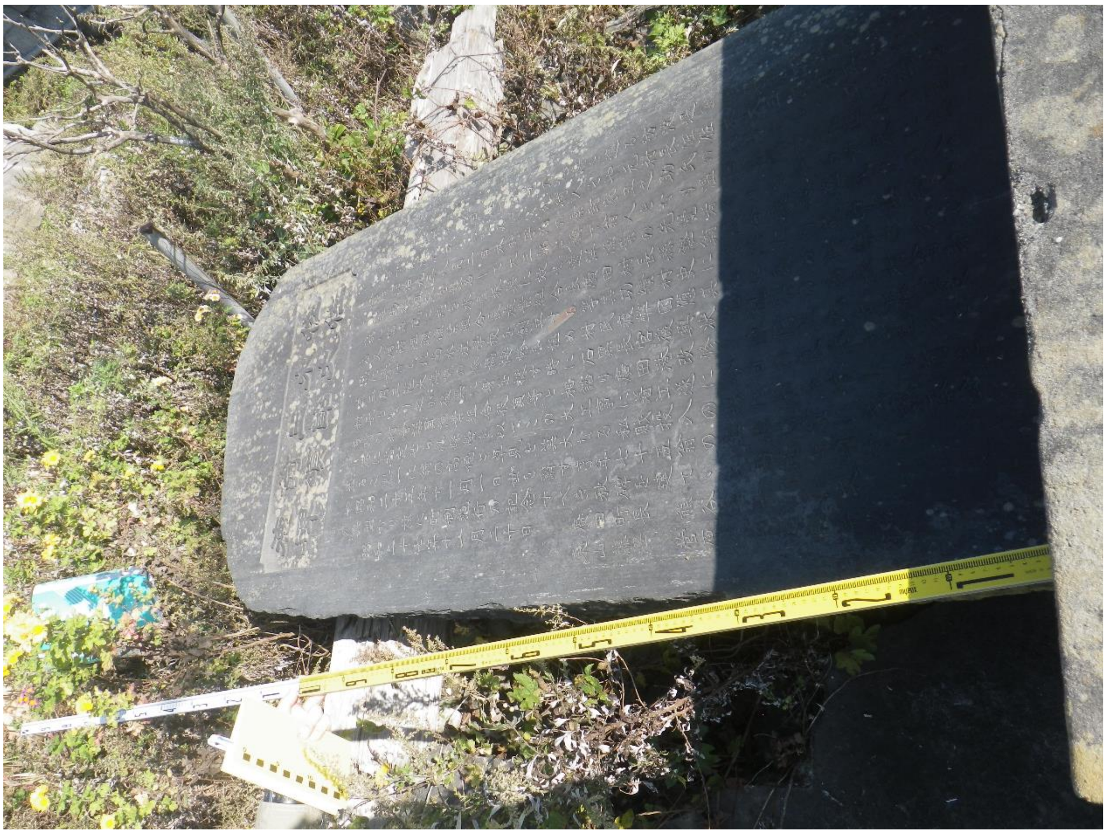

Fig.S18

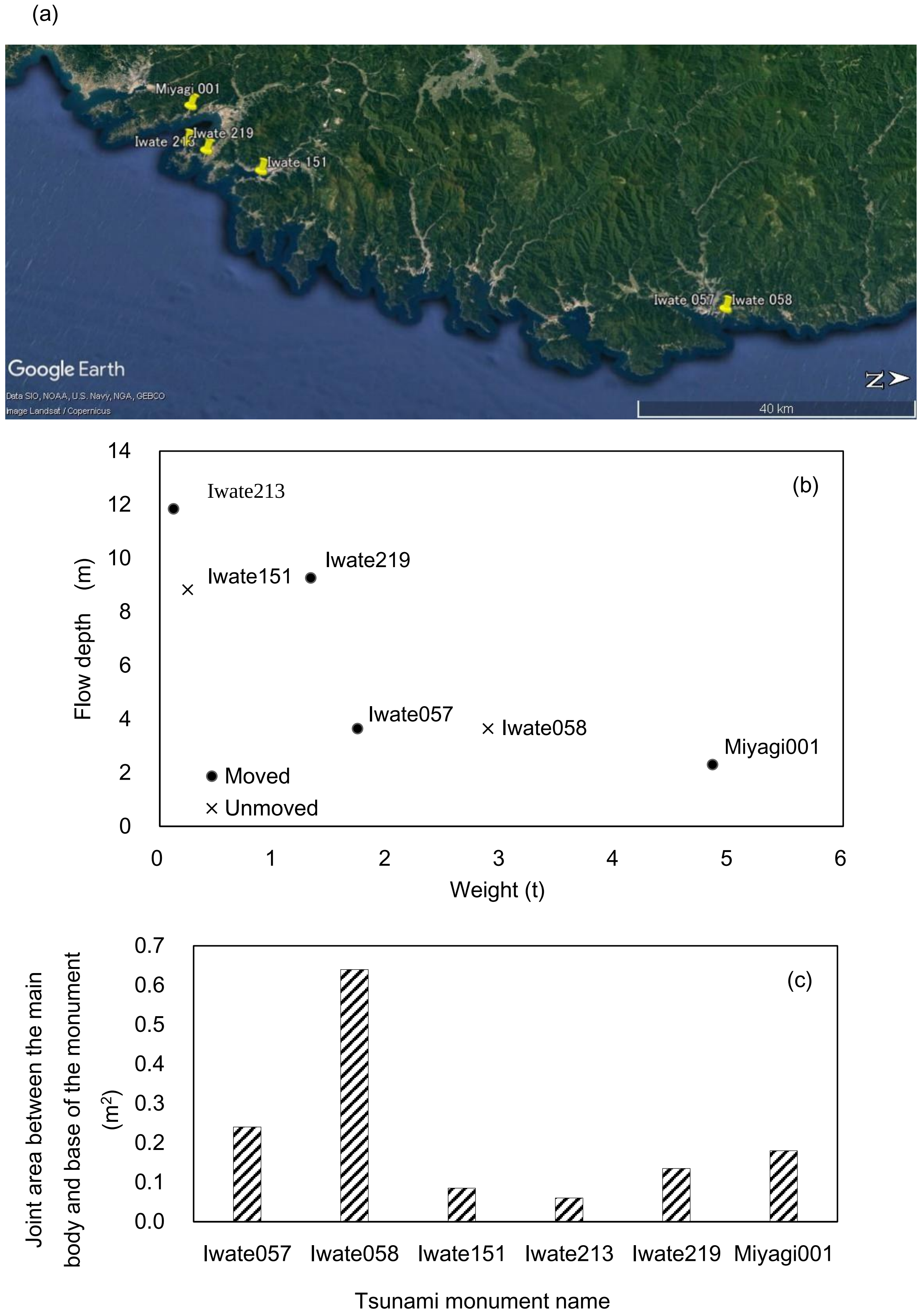

(a)
Miyagi 001
Iwate 219
Iwate 213
Iwate 151
Iwate 057
Iwate 058
Google Earth
Data SIO, NOAA, U.S. Navy, NGA, GEBCO
Image Landsat / Copernicus
40 km
N
(b)
Flow depth (m)
Weight (t)
0
2
4
6
8
10
12
14
0
1
2
3
4
5
6
Iwate213
Iwate151
Iwate219
Iwate057
Iwate058
Miyagi001
Moved
Unmoved
(c)
Joint area between the main body and base of the monument (m2)
0.0
0.1
0.2
0.3
0.4
0.5
0.6
0.7
Iwate057
Iwate058
Iwate151
Iwate213
Iwate219
Miyagi001
Tsunami monument name
